# Supplementary material for: Chemotherapy and Heart-Specific Mortality in Elderly Men with Prostate Cancer: A Propensity Score Matching Analysis
Source: PLoS One. 2025 Apr 11;20(4):e0318429. doi: 10.1371/journal.pone.0318429 (PMC11990641; doi:10.1371/journal.pone.0318429)
Supplement: S3 File — (PDF) [file pone.0318429.s003.pdf]

SEER RESEARCH PLUS DATA DESCRIPTION

CASES DIAGNOSED IN 1975-2019*

Submission: *November 2021*

Follow-up Cutoff Date: *December 31, 2019*

Documentation Version: *April 2022*

Diagnosis Years: *1975-2019*

TABLE OF CONTENTS

PATIENT ID 6
SEER REGISTRY 6
SEER REGISTRY (WITH CA AND GA AS WHOLE STATES) 7
LOUISIANA 2005 – 1ST VS 2ND HALF OF THE YEAR 8
MARITAL STATUS AT DIAGNOSIS 8
RACE/ETHNICITY 9
SEX 10
AGE RECODE WITH SINGLE AGES AND 100+ 10
AGE RECODE WITH SINGLE AGES AND 85+ 10
SEQUENCE NUMBER 11
MONTH OF DIAGNOSIS 12
YEAR OF DIAGNOSIS 12
COC ACCREDITED FLAG (2018+) 12
MONTH OF DIAGNOSIS RECODE 13
PRIMARY SITE 13
PRIMARY SITE - LABELED 14
LATERALITY 14
HISTOLOGY ICD-O-2 15
BEHAVIOR CODE ICD-O-2 17
HISTOLOGIC TYPE ICD-O-3 17
BEHAVIOR CODE ICD-O-3 18
ICD-O-3 HIST/BEHAV 18
ICD-O-3 HIST/BEHAV, MALIGNANT 18
GRADE (THRU 2017) 19
SCHEMA ID (2018+) 19
GRADE CLINICAL (2018+) 19
GRADE PATHOLOGICAL (2018+) 20
DIAGNOSTIC CONFIRMATION 20
TYPE OF REPORTING SOURCE 21
EOD 10— SIZE (1988-2003) 22
EOD 10—EXTENT (1988-2003) 22
EOD 10—PROSTATE PATH EXT (1995-2003) 22
EOD 10—NODES (1995-2003) 23
REGIONAL NODES POSITIVE (1988+) 23
REGIONAL NODES EXAMINED (1988+) 24
EXPANDED EOD (1) – EXPANDED EOD (13) 24
2-DIGIT NS EOD / 2-DIGIT SS EOD1973-1982 25
EOD 4 – SIZE (1983-1987) 25
EOD 4 – EXTENT (1983-1987) 25
EOD 4 – NODES (1983-1987) 25
CODING SYSTEM – EOD (1973-2003) 26
TUMOR MARKER 1 (1990-2003) 26
TUMOR MARKER 2 (1990-2003) 28
TUMOR MARKER 3 (1998-2003) 29
CS TUMOR SIZE (2004-2015) 30
CS EXTENSION (2004-2015) 30
CS LYMPH NODES (2004-2015) 31
CS METS AT DX (2004-2015) 31
CS SITE-SPECIFIC FACTOR 1 (2004-2017 VARYING BY SCHEMA) 32
CS SITE-SPECIFIC FACTOR 2 (2004-2017 VARYING BY SCHEMA) 32
CS SITE-SPECIFIC FACTOR 3 (2004-2017 VARYING BY SCHEMA) 32
CS SITE-SPECIFIC FACTOR 4 (2004-2017 VARYING BY SCHEMA) 33

CS SITE-SPECIFIC FACTOR 5 (2004-2017 VARYING BY SCHEMA) 33
CS SITE-SPECIFIC FACTOR 6 (2004-2017 VARYING BY SCHEMA) 33
CS SITE-SPECIFIC FACTOR 7 (2004-2017 VARYING BY SCHEMA) 34
CS SITE-SPECIFIC FACTOR 8 (2004-2017 VARYING BY SCHEMA) 34
CS SITE-SPECIFIC FACTOR 9 (2004-2017 VARYING BY SCHEMA) 34
CS SITE-SPECIFIC FACTOR 10 (2004-2017 VARYING BY SCHEMA) 35
CS SITE-SPECIFIC FACTOR 11 (2004-2017 VARYING BY SCHEMA) 35
CS SITE-SPECIFIC FACTOR 12 (2004-2017 VARYING BY SCHEMA) 35
CS SITE-SPECIFIC FACTOR 13 (2004-2017 VARYING BY SCHEMA) 36
CS SITE-SPECIFIC FACTOR 15 (2004-2017 VARYING BY SCHEMA) 36
CS SITE-SPECIFIC FACTOR 16 (2004-2017 VARYING BY SCHEMA) 36
CS SITE-SPECIFIC FACTOR 25 (2004-2017 VARYING BY SCHEMA) 37
DERIVED AJCC T, 6TH ED (2004-2015) 38
DERIVED AJCC N, 6TH ED (2004-2015) 39
DERIVED AJCC M, 6TH ED (2004-2015) 40
DERIVED AJCC STAGE GROUP, 6TH ED (2004-2015) 40
SUMMARY STAGE 2000 (1998-2017) 42
COMBINED SUMMARY STAGE (2004+) 42
CS VERSION INPUT ORIGINAL (2004-2015) 42
CS VERSION DERIVED (2004-2015) 43
CS VERSION INPUT CURRENT (2004-2015) 43
RX SUMM—SURG PRIM SITE (1998+) 44
RX SUMM—SCOPE REG LN SUR (2003+) 45
RX SUMM-SURG OTH REG/DIS (2003+) 46
RX SUMM-REG LN EXAMINED (1998-2002) 46
RX SUMM-SYSTEMIC SUR SEQ 47
RX SUMM--SURG/RAD SEQ 47
REASON NO CANCER-DIRECTED SURGERY 48
RADIATION RECODE 49
CHEMOTHERAPY RECODE (YES, NO/UNK) 49
SITE SPECIFIC SURGERY (1973-1997 VARYING DETAIL BY YEAR AND SITE) 50
SCOPE OF REG LYMPH ND SURG (1998-2002) 50
SURGERY OF OTH REG/DIS SITES (1998-2002) 51
RECORD NUMBER RECODE 51
AGE RECODE WITH <1 YEAR OLDS 52
SITE RECODE ICD-O-3/WHO 2008 53
SITE RECODE ICD-O-3/WHO 2008 (FOR SIRS) 53
SITE RECODE – RARE TUMORS 53
BEHAVIOR RECODE FOR ANALYSIS 54
HISTOLOGY RECODE—BROAD GROUPINGS 54
HISTOLOGY RECODE—BRAIN GROUPINGS 57
ICCC SITE RECODE EXTENDED 3RD EDITION/IARC 2017 58
TNM 7/CS V0204+ SCHEMA (THRU 2017) 58
TNM 7/CS V0204+ SCHEMA RECODE 63
RACE RECODE (WHITE, BLACK, OTHER) 64
RACE RECODE (W, B, AI, API) 64
ORIGIN RECODE NHIA (HISPANIC, NON-HISP) 65
RACE AND ORIGIN RECODE (NHW, NHB, NHAIAN, NHAPI, HISPANIC) 65
SEER HISTORIC STAGE A (1973-2015) 66
AJCC STAGE 3RD EDITION (1988-2003) 66
SEER MODIFIED AJCC STAGE 3RD ED (1988-2003) 67
FIRST MALIGNANT PRIMARY INDICATOR 67

STATE-COUNTY ... 67
COUNTY ... 68
COUNTY ATRRIBUTES ... 68
PRCDA 2017 ... 68
PRCDA REGION ... 69
COD TO SITE RECODE ... 69
COD TO SITE REC KM ... 70
VITAL STATUS RECODE (STUDY CUTOFF USED) ... 70
IHS LINK ... 70
SUMMARY STAGE 2000 (1998-2017) ... 71
AYA SITE RECODE/WHO 2008 ... 71
AYA SITE RECODE 2020 REVISION ... 71
LYMPHOID NEOPLASM RECODE 2021 REVISION ... 72
LYMPHOMA SUBTYPE RECODE/WHO 2008 (THRU 2017) ... 72
SEER BRAIN AND CNS RECODE ... 73
ICCC SITE RECODE 3RD EDITION/IARC 2017 ... 73
SEER CAUSE-SPECIFIC DEATH CLASSIFICATION ... 73
SEER OTHER CAUSE OF DEATH CLASSIFICATION ... 74
CS TUMOR SIZE/EXT EVAL (2004-2015) ... 74
CS REG NODE EVAL (2004-2015) ... 74
CS METS EVAL (2004-2015) ... 75
PRIMARY BY INTERNATIONAL RULES ... 75
ER STATUS RECODE BREAST CANCER (1990+) ... 75
PR STATUS RECODE BREAST CANCER (1990+) ... 76
CS SCHEMA—AJCC 6TH EDITION ... 76
LYMPH-VASCULAR INVASION (2004+ VARYING BY SCHEMA) ... 79
SURVIVAL MONTHS ... 79
SURVIVAL MONTHS FLAG ... 80
DERIVED AJCC T, 7TH ED (2010-2015) ... 81
DERIVED AJCC N, 7TH ED (2010-2015) ... 82
DERIVED AJCC M, 7TH ED (2010-2015) ... 83
DERIVED AJCC STAGE GROUP, 7TH ED (2010-2015) ... 84
BREAST—ADJUSTED AJCC 6TH T (1988-2015) ... 86
BREAST—ADJUSTED AJCC 6TH N (1988-2015) ... 87
BREAST—ADJUSTED AJCC 6TH M (1988-2015) ... 88
BREAST—ADJUSTED AJCC 6TH STAGE (1988-2015) ... 89
DERIVED HER2 RECODE (2010+) ... 90
BREAST SUBTYPE (2010+) ... 91
LYMPHOMAS: ANN ARBOR STAGING (1983-2015) ... 91
SEER COMBINED METS AT DX-BONE (2010+) ... 92
SEER COMBINED METS AT DX-BRAIN (2010+) ... 92
SEER COMBINED METS AT DX-LIVER (2010+) ... 93
SEER COMBINED METS AT DX-LUNG (2010+) ... 93
T VALUE - BASED ON AJCC 3RD (1988-2003) ... 94
N VALUE - BASED ON AJCC 3RD (1988-2003) ... 94
M VALUE - BASED ON AJCC 3RD (1988-2003) ... 94
TOTAL NUMBER OF IN SITU/MALIGNANT TUMORS FOR PATIENT ... 95
TOTAL NUMBER OF BENIGN/BORDERLINE TUMORS FOR PATIENT ... 95
RADIATION TO BRAIN OR CNS RECODE (1988-1997) ... 95
TUMOR SIZE SUMMARY (2016+) ... 95
DERIVED SEER CMB STG GRP (2016-2017) ... 96
DERIVED SEER COMBINED T (2016-2017) ... 96

DERIVED SEER COMBINED N (2016-2017) 97
DERIVED SEER COMBINED M (2016-2017)........ 97
DERIVED SEER COMBINED T SRC (2016-2017) 97
DERIVED SEER COMBINED N SRC (2016-2017)........ 98
DERIVED SEER COMBINED M SRC (2016-2017) 98
TNM EDITION NUMBER (2016-2017) 99
METS AT DX—DISTANT LN (2016+)........ 99
METS AT DX—OTHER (2016+)........ 100
AJCC ID (2018+) 100
EOD SCHEMA ID RECODE (2010+)........ 101
DERIVED EOD 2018 T (2018+)........ 101
DERIVED EOD 2018 N (2018+) 101
DERIVED EOD 2018 M (2018+)........ 101
DERIVED EOD 2018 STAGE GROUP (2018+) 102
EOD PRIMARY TUMOR (2018+)........ 102
EOD REGIONAL NODES (2018+)........ 102
EOD METS (2018+)........ 102
MONTHS FROM DIAGNOSIS TO TREATMENT........ 103

PATIENT ID

NAACCR Item #: 20
SAS Variable Name: Patient_ID
Research: Yes
Research Limited-Field: Yes
Research Plus Limited-Field: Yes

Field Description: This field is used to uniquely identify a person. One person can have multiple primaries but the same Patient ID. See the sequence number for more information about the primary. This is a dummy number and is not the number used by the registry to identify the patient. The same number is not used across all submissions for each patient.

SEER REGISTRY

NAACCR Item #: 40
SAS Variable Name: N/A
Research: No
Research Limited-Field: No
Research Plus Limited-Field: Yes

Field Description: A unique code assigned to each participating SEER registry. The number identifies the registry sending the record and what population the data are based on.

Note: This data item is not available at the individual level.

Code	Description
0000001501	San Francisco-Oakland SMSA (1975+)
0000001502	Connecticut (1975+)
0000001521	Hawaii (1975+)
0000001522	Iowa (1975+)
0000001523	New Mexico (1975+)
0000001525	Seattle (Puget Sound) (1975+)
0000001526	Utah (1975+)
0000001527	Metropolitan Atlanta (1975+)
0000001529	Alaska (1992+)
0000001531	San Jose-Monterey (1992+)
0000001535	Los Angeles (1992+)
0000001537	Rural Georgia (1992+)
0000001541	Greater California (excl. SF, Los Ang. & SJ) (2000+)
0000001542	Kentucky (2000+)
0000001543	Louisiana (2000+)
0000001544	New Jersey (2000+)
0000001547	Greater Georgia (excluding AT and RG)

(Continued from SEER REGISTRY)

Code	Description
0000001561	Idaho* (2000+)
0000001562	New York* (2000+)
0000001563	Massachusetts* (2000+)
0000001565	Illinois* (2000+)
0000001566	Texas* (2000+)

(Year in parentheses refers to first diagnosis year of data available)

*Note: Registry data only available in limited-field databases.

SEER REGISTRY (WITH CA AND GA AS WHOLE STATES)

NAACCR Item #: N/A
SAS Variable Name: SEER_registry_with_CA_and_GA_as_whole_states
Research: No
Research Limited-Field: No
Research Plus Limited-Field: Yes

Field Description: SEER registries from California and Georgia are combined into state-level groupings representing those two entire states. This is the only geographic indicator available for analysis at the individual level.

Code	Description
02	Alaska Natives
06	California
09	Connecticut
13	Georgia
15	Hawaii
16	Idaho
19	Iowa
21	Kentucky
22	Louisiana
25	Massachusetts
34	New Jersey
35	New Mexico
36	New York
49	Utah
53	Seattle (Puget Sound)
65	Illinois
66	Texas

LOUISIANA 2005 – 1ST VS 2ND HALF OF THE YEAR

NAACCR Item #: N/A
SAS Variable Name: N/A
Research: No
Research Limited-Field: No
Research Plus Limited-Field: Yes

Field Description: This variable can be used to distinguish cases diagnosed in Louisiana between July and December of 2005, when reporting may have been affected by hurricanes Katrina and Rita.

Note: This data item is not available at the individual level.

Code	Description
0	Not applicable (not 2005 Louisiana)
1	Louisiana: January-June (includes unknown) 2005
2	Louisiana: July-December 2005

MARITAL STATUS AT DIAGNOSIS

NAACCR Item #: 150
SAS Variable Name: Marital_status_at_diagnosis
Research: No
Research Limited-Field: No
Research Plus Limited-Field: No

Field Description: This data item identifies the patient's marital status at the time of diagnosis for the reportable tumor.

Code	Description
1	Single (never married)
2	Married (including common law)
3	Separated
4	Divorced
5	Widowed
6	Unmarried or domestic partner (same sex or opposite sex or unregistered)
9	Unknown
14	Blank

RACE/ETHNICITY

NAACCR Item #: N/A
SAS Variable Name: Race_ethnicity
Research: Yes
Research Limited-Field: Yes
Research Plus Limited-Field: Yes

Field Description: Recode which gives priority to non-white races for persons of mixed races. Note that not all codes were in effect for all years.

Code	Description
01	White
02	Black
03	American Indian, Aleutian, Alaskan Native or Eskimo (includes all indigenous populations of the Western hemisphere)
04	Chinese
05	Japanese
06	Filipino
07	Hawaiian
08	Korean (Effective with 1/1/1988 dx)
10	Vietnamese (Effective with 1/1/1988 dx)
11	Laotian (Effective with 1/1/1988 dx)
12	Hmong (Effective with 1/1/1988 dx)
13	Kampuchean (including Khmer and Cambodian) (Effective with 1/1/1988 dx)
14	Thai (Effective with 1/1/1994 dx)
15	Asian Indian or Pakistani, NOS (Effective with 1/1/1988 dx)
16	Asian Indian (Effective with 1/1/2010 dx)
17	Pakistani (Effective with 1/1/2010 dx)
20	Micronesian, NOS (Effective with 1/1/1991)
21	Chamorran (Effective with 1/1/1991 dx)
22	Guamanian, NOS (Effective with 1/1/1991 dx)
25	Polynesian, NOS (Effective with 1/1/1991 dx)
26	Tahitian (Effective with 1/1/1991 dx)
27	Samoan (Effective with 1/1/1991 dx)
28	Tongan (Effective with 1/1/1991 dx)
30	Melanesian, NOS (Effective with 1/1/1991 dx)
31	Fiji Islander (Effective with 1/1/1991 dx)
32	New Guinean (Effective with 1/1/1991 dx)
96	Other Asian, including Asian, NOS and Oriental, NOS (Effective with 1/1/1991 dx)
97	Pacific Islander, NOS (Effective with 1/1/1991 dx)
98	Other
99	Unknown

SEER Participants San Francisco, San Jose-Monterey, and Los Angeles are permitted to use codes 14 and 20-97 for cases diagnosed after January 1, 1987. Greater California is permitted to use codes 14 and 20-97 for cases diagnosed after January 1, 1988. Other SEER participants may choose to recode cases diagnosed prior to 1991 using 14 and 20-97 if all cases in the following race codes are reviewed: 96 Other Asian; 97 Pacific Islander, NOS; 98 Other; and 99 unknown.

SEX

NAACCR Item #: 220
SAS Variable Name: Sex
Research: Yes
Research Limited-Field: Yes
Research Plus Limited-Field: Yes

Field Description: This data item identifies the sex of the patient at diagnosis.

Code	Description
1	Male
2	Female
9	Not stated (unknown)

AGE RECODE WITH SINGLE AGES AND 100+

NAACCR Item #: N/A
SAS Variable Name: Agerecodewithsingleages_and_100
Research: No
Research Limited-Field: No
Research Plus Limited-Field: Yes

Field Description: This data item represents the age of the patient at diagnosis **for this cancer**. The code is three digits and represents the patient's actual age in years up to age 99. Age 100 and over are grouped as 100+.

Code	Description
000-099	Actual age in years
100	100+ years
127	Unknown age

AGE RECODE WITH SINGLE AGES AND 85+

NAACCR Item #: N/A
SAS Variable Name: Agerecodewithsingleages_and_85
Research: Yes
Research Limited-Field: Yes
Research Plus Limited-Field: Yes

Field Description: This data item represents the age of the patient at diagnosis **for this cancer**. The code is three digits and represents the patient's actual age in years up to age 84. Age 85 and over are grouped as 85+.

Code	Description
000-084	Actual age in years
85	85+ years
127	Unknown age

SEQUENCE NUMBER

NAACCR Item #: 380
SAS Variable Name: Sequence_number
Research: Yes
Research Limited-Field: Yes
Research Plus Limited-Field: Yes

Field Description: Sequence Number-Central describes the number and sequence of all reportable malignant, in situ, benign, and borderline primary tumors, which occur over the lifetime of a patient. The sequence number may change over the lifetime of the patient. If an individual previously diagnosed with a single reportable malignant neoplasm is subsequently diagnosed with a second reportable malignant neoplasm, the sequence code for the first neoplasm changes from 00 to 01.

This sequence number counts all tumors that were reportable in the year they were diagnosed even if the tumors occurred before the registry existed, or before the registry participated in the SEER Program. The purpose of sequencing based on the patient's *lifetime* is to truly identify the patients for survival analysis who only had one malignant primary in their lifetimes.

In Situ/Malignant as Federally Required based on Diagnosis Year

Code	Description
00	One primary only in the patient's lifetime
01	First of two or more primaries
02	Second of two or more primaries
..	(Actual number of this primary)
59	Fifty-ninth of fifty-nine or more primaries
99	Unspecified or unknown sequence number of Federally required in situ or malignant tumors. Sequence number 99 can be used if there is a malignant tumor and its sequence number is unknown. (If there is known to be more than one malignant tumor, then the tumors must be sequenced.)

Non-malignant Tumor as Federally Required based on Diagnosis Year

Code	Description
60	Only one non-malignant tumor or central registry-defined neoplasm
61	First of two or more non-malignant tumors or central registry-defined neoplasms
62	Second of two or more non-malignant tumors or central registry-defined neoplasms
..	..
87	Twenty-seventh of twenty-seven
88	Unspecified or unknown sequence number of non-malignant tumor or central-registry defined neoplasms. (Sequence number 88 can be used if there is a non-malignant tumor and its sequence number is unknown. If there is known to be more than one non-malignant tumor, then the tumors must be sequenced.)

MONTH OF DIAGNOSIS

NAACCR Item #: 390
SAS Variable Name: N/A
Research: No
Research Limited-Field: No
Research Plus Limited-Field: Yes

Field Description: The month of diagnosis is the month the tumor was first diagnosed by a recognized medical practitioner, whether clinically or microscopically confirmed.

Note: This data item is not available at the individual level.

Allowable values
MM = 01 – 12
14=Blank

YEAR OF DIAGNOSIS

NAACCR Item #: 390
SAS Variable Name: Year_of_diagnosis
Research: Yes
Research Limited-Field: Yes
Research Plus Limited-Field: Yes

Field Description: The year of diagnosis is the year the tumor was first diagnosed by a recognized medical practitioner, whether clinically or microscopically confirmed.

Allowable values
YYYY = 1975 – forward

COC ACCREDITED FLAG (2018+)

NAACCR Item #: 2152
SAS Variable Name: CoC_Accredited_Flag_2018
Research: Yes
Research Limited-Field: NYeso
Research Plus Limited-Field: Yes

Field Description: CoC Accredited Flag is assigned at the point and time of data abstraction to label an abstract being prepared for an analytic cancer case at a facility accredited by the Commission on Cancer (CoC). The flag may be assigned manually or can be defaulted by the registry's software.

MONTH OF DIAGNOSIS RECODE

NAACCR Item #: N/A
SAS Variable Name: N/A
Research: No
Research Limited-Field: No
Research Plus Limited-Field: Yes

Field Description: This field uses other known data points to input unknown month of diagnosis.

Note: This data item is not available at the individual level.

Code	Description
1	January
2	February
3	March
4	April
5	May
6	June
7	July
8	August
9	September
10	October
11	November
12	December

PRIMARY SITE

NAACCR Item #: 400
SAS Variable Name: Primary_Site
Research: Yes
Research Limited-Field: Yes
Research Plus Limited-Field: Yes

Field Description: This data item identifies the site in which the primary tumor originated. See the *International Classification of Diseases for Oncology,* Third Edition (ICD-O-3) for topography codes. The decimal point is eliminated.

Cases diagnosed 1977-1991 were coded using the *International Classification of Diseases for Oncology*, 1976 Edition (ICD-O-1976). Prior to 1977 diagnoses, cases were coded using the *Manual of Tumor Nomenclature and Coding*, 1968 (MOTNAC).

All cases 1975-1991 were machine-converted to ICD-O-2 codes without complete hand review.

PRIMARY SITE - LABELED

NAACCR Item #: 400
SAS Variable Name: Primary_Site_labeled
Research: Yes
Research Limited-Field: Yes
Research Plus Limited-Field: Yes

Field Description: This provides the primary site code in ICD-O-3 and a descriptive primary site label. Note that the label is the preferred ICD-O-3 bolded name and there may be other sites or sub-sites included in the code but not reflected in the preferred term. Refer to ICD-O-3 for further information. Cases with years of diagnosis before 1992 were converted to ICD-O-3 from earlier versions.

LATERALITY

NAACCR Item #: 410
SAS Variable Name: Laterality
Research: Yes
Research Limited-Field: Yes
Research Plus Limited-Field: Yes

Field Description: Laterality describes the side of a paired organ or side of the body on which the reportable tumor originated. Starting with cases diagnosed January 1, 2004 and later, laterality is coded for select invasive, benign, and borderline primary intracranial and CNS tumors.

See http://seer.cancer.gov/manuals/primsite.laterality.pdf for a list of sites for which SEER requires information on laterality.

Code	Description
0	Not a paired site
1	Right: origin of primary
2	Left: origin of primary
3	Only one side involved, right or left origin unspecified
4	Bilateral involvement, lateral origin unknown; stated to be single primary<br>• Both ovaries involved simultaneously, single histology<br>• Bilateral retinoblastomas<br>• Bilateral Wilms's tumors
5	Paired site: midline tumor
9	Paired site, but no information concerning laterality; midline tumor

HISTOLOGY ICD-O-2

NAACCR Item #: 420
SAS Variable Name: Histology_ICD_O_2
Research: Yes
Research Limited-Field: No
Research Plus Limited-Field: No

Field Description: NOTE: ALL CASES DIAGNOSED 1975-91 HAVE BEEN CONVERTED TO THE *INTERNATIONAL CLASSIFICATION OF DISEASES FOR ONCOLOGY*, SECOND EDITION (ICD-O-2, 1992).

The SEER program has used several different, but related, coding systems for morphology over time. One should be extremely careful when doing any analysis of trends related to morphology. It is suggested that these analyses start with diagnoses no earlier than 1977 and that special attention be paid to the changes for 1986-91 cases due to the use of the *International Classification of Disease for Oncology, Field Trial Edition, March 1988* and for 1992+ cases due to the use of ICD-O-2.

Analyses of morphology data are usually limited to microscopically confirmed cases only.

HISTOLOGY CODING

FOR CASES DIAGNOSED 1975-85:

Histologic types are defined in the morphology section of the *International Classification of Diseases for Oncology*, 1976 Edition (ICD-O, 1976). However prior to the introduction of ICD-O, 1976, morphology was coded using the *Manual of Tumor Nomenclature and Coding*, 1968 (MOTNAC). With the introduction of ICD-O, 1976, all cases previously coded using MOTNAC were machine converted without hand review using the conversion table, *Conversion of Morphology Sections (neoplasms) of 1968 Manual of Tumor Nomenclature and Coding (MOTNAC) and 1965 Systematized Nomenclature of Pathology (SNOP) to 1976 International Classification of Diseases for Oncology*, developed by Constance Percy. The morphology codes of ICD-O, 1976, are more specific than those of MOTNAC. Thus, less detailed information on morphology is available for cases diagnosed 1975-76. For example, when using MOTNAC large cell carcinoma is included with carcinoma, not otherwise specified (NOS); when using ICD-O, 1976, large cell carcinoma is a separate entity having its own code. All have been machine converted to ICD-O-2.

(Continued on next page)

(Continued from HISTOLOGY ICD-O-2)

FOR CASES DIAGNOSED 1975-85:

In addition, the following special morphology codes were used by the SEER Program:

Breast — All years

Code	Description
8522/3	Infiltrating duct carcinoma and lobular carcinoma
8523/3	Infiltrating duct carcinoma and lobular carcinoma in situ
8524/3	Intraductal carcinoma and lobular carcinoma
8522/2	Intraductal carcinoma and lobular carcinoma in situ
8543/3	Paget's disease with intraductal carcinoma

All sites — Introduced for coding in approximately 1982

Code	Description
9616/3	Lennert's lymphoma
9624/3	Malignant lymphoma, large cell, cleaved, diffuse
9634/3	Malignant lymphoma, large cell, non-cleaved, diffuse
9723/3	True histiocytic lymphoma

FOR CASES DIAGNOSED 1986-91:

Histologic types are defined in the morphology section of the *International Classification of Diseases for Oncology, Field Trial Edition, 1986* (ICD-O FT). (This volume is no longer available. It was replaced by the *International Classification of Diseases for Oncology, Field Trial Edition, March 1988* (ICD-O FT 1988). Pages i-vi, 23 of the latter volume list the differences between the two Field Trials, present a summary of the changes to ICD-O, 1976, and define the symbols used in the morphology section. These were converted to ICD-O-2.

FOR CASES DIAGNOSED 1992-2000:

Histologic types are defined in the morphology section of ICD-O-2. See pages 137+ in ICD-O-2 for additions and changes.

FOR CASES DIAGNOSED AFTER 2000:

All cases diagnosed after 2000 were converted from ICD-O-3 to ICD-O-2.

BEHAVIOR CODE ICD-O-2

NAACCR Item #: 430
SAS Variable Name: Behavior_code_ICD_O_2
Research: Yes
Research Limited-Field: No
Research Plus Limited-Field: No

Field Description: Behavior codes are also defined in ICD-O-2, 1992.

Note: For bladder only, all in situs (/2) are converted to invasives (/3) before inclusion on this file.

Cervix in situ not required.

FOR CASES DIAGNOSED AFTER 2000:

All cases diagnosed after 2000 were converted from ICD-O-3 to ICD-O2.

HISTOLOGIC TYPE ICD-O-3

NAACCR Item #: 522
SAS Variable Name: Histologic_Type_ICD_O_3
Research: Yes
Research Limited-Field: Yes
Research Plus Limited-Field: Yes

Field Description: The data item Histologic Type describes the microscopic composition of cells and/or tissue for a specific primary. The tumor type or histology is a basis for staging and determination of treatment options. It affects the prognosis and course of the disease. The *International Classification of Diseases for Oncology*, Third Edition (ICD-O-3) is the standard reference for coding the histology for tumors diagnosed in 2001 and later.

All ICD-O-2 histologies for 1975-2000 were converted to ICD-O-3.

BEHAVIOR CODE ICD-O-3

NAACCR Item #: 523
SAS Variable Name: Behavior_code_ICD_O_3
Research: Yes
Research Limited-Field: Yes
Research Plus Limited-Field: Yes

Field Description: SEER requires registries to collect malignancies with in situ /2 and malignant /3 behavior codes as described in ICD-O-3. SEER requires registries to collect benign /0 and borderline /1 intracranial and CNS tumors for cases diagnosed on or after 1/1/2004. Behavior is the fifth digit of the morphology code after the slash (/). See ICD-O-3 (page 66) for a discussion of the behavior code.

Code	Description
0	Benign (Reportable for intracranial and CNS sites only)
1	Uncertain whether benign or malignant, borderline malignancy, low malignant potential, and uncertain malignant potential (Reportable for intracranial and CNS sites only)
2	Carcinoma in situ; intraepithelial; noninfiltrating; noninvasive
3	Malignant, primary site (invasive)

All ICD-O-2 behaviors for 1975-2000 were converted to ICD-O-3.

ICD-O-3 HIST/BEHAV

NAACCR Item #: N/A
SAS Variable Name: ICD_O_3_Hist_behav
Research: Yes
Research Limited-Field: Yes
Research Plus Limited-Field: Yes

Field Description: Labeled version of ICD-O-3 values for all behaviors. See SEER*Stat dictionary for labels.

ICD-O-3 HIST/BEHAV, MALIGNANT

NAACCR Item #: N/A
SAS Variable Name: ICD_O_3_Hist_behav_malignant
Research: Yes
Research Limited-Field: Yes
Research Plus Limited-Field: Yes

Field Description: Labeled version of ICD-O-3 values for malignant tumors. All non-malignant tumors are grouped into one value. See SEER*Stat dictionary for labels.

GRADE (THRU 2017)

NAACCR Item #: 440
SAS Variable Name: Grade_thru_2017
Research: Yes
Research Limited-Field: Yes
Research Plus Limited-Field: Yes

Field Description: Grading and differentiation codes of 1-4, 9 are defined in ICD-O-2; 1992. Grade information may be incomplete for cases diagnosed before 1977.

In the early 1980's, additional codes specifying T-cell, B-cell, or null cell involvement in lymphomas and leukemias (histologies M9590-9940) were introduced by SEER. Because the reporting requirements and medical terminology have changed over time, care should be exercised when analyzing this information.

Code	Description
1	Grade I; grade i; grade 1; well differentiated; differentiated, NOS
2	Grade II; grade ii; grade 2; moderately differentiated; moderately differentiated; intermediate differentiation
3	Grade III; grade iii; grade 3; poorly differentiated; differentiated
4	Grade IV; grade iv; grade 4; undifferentiated; anaplastic
5	T-cell; T-precursor
6	B-cell; Pre-B; B-Precursor
7	Null cell; Non T-non B;
8	N K cell (natural killer cell)
9	cell type not determined, not stated or not applicable

SCHEMA ID (2018+)

NAACCR Item #: 3800
SAS Variable Name: Schema_ID_2018
Research: Yes
Research Limited-Field: Yes
Research Plus Limited-Field: Yes

Field Description: This variable allows the linkage of 2018+ Site-Specific Data Items (SSDIs), including grade data items, with the appropriate site/histology grouping.

GRADE CLINICAL (2018+)

NAACCR Item #: 3843
SAS Variable Name: Grade_Clinical_2018
Research: Yes
Research Limited-Field: Yes
Research Plus Limited-Field: Yes

Field Description: Refer to the most recent Grade Manual for additional site-specific instructions. https://apps.naaccr.org/ssdi/list/

GRADE PATHOLOGICAL (2018+)

NAACCR Item #: 3844
SAS Variable Name: Grade_Pathological_2018
Research: Yes
Research Limited-Field: Yes
Research Plus Limited-Field: Yes

Field Description: Refer to the most recent Grade Manual for additional site-specific instructions. https://apps.naaccr.org/ssdi/list/

DIAGNOSTIC CONFIRMATION

NAACCR Item #: 490
SAS Variable Name: Diagnostic_Confirmation
Research: Yes
Research Limited-Field: Yes
Research Plus Limited-Field: Yes

Field Description: This data item records the best method used to confirm the presence of the cancer being reported. The data item is not limited to the confirmation at the time of diagnosis; it is the best method of confirmation during the entire course of the disease.

Microscopically Confirmed

Code	Description
1	Positive histology
2	Positive cytology
3	Positive histology PLUS - positive immunophenotyping AND/OR positive genetic studies (Used only for hematopoietic and lymphoid neoplasms M-9590/3-9992/3)
4	Positive microscopic confirmation, method not specified

(Continued on next page)

(Continued from DIAGNOSTIC CONFIRMATION)

Not Microscopically Confirmed

Code	Description
5	Positive laboratory test/marker study
6	Direct visualization without microscopic confirmation
7	Radiology and other imaging techniques without microscopic confirmation
8	Clinical diagnosis only (other than 5, 6, or 7)

Confirmation Unknown

Code	Description
9	Unknown whether microscopically confirmed; death certificate only

TYPE OF REPORTING SOURCE

NAACCR Item #: 500
SAS Variable Name: Type_of_Reporting_Source
Research: Yes
Research Limited-Field: Yes
Research Plus Limited-Field: Yes

Field Description: The Type of Reporting Source identifies the source documents used to abstract the case. This is not necessarily the original document that identified the case; rather, it is the source that provided the best information.

Code	Description
1	Hospital inpatient; Managed health plans with comprehensive, unified medical records (new code definition effective with diagnosis on or after 1/1/2006)
2	Radiation Treatment Centers or Medical Oncology Centers (hospital-affiliated or independent) (effective with diagnosis on or after 1/1/2006)
3	Laboratory Only (hospital-affiliated or independent)
4	Physician’s Office/Private Medical Practitioner (LMD)
5	Nursing/Convalescent Home/Hospice
6	Autopsy Only
7	Death Certificate Only
8	Other hospital outpatient units/surgery centers (effective with diagnosis on or after 1/1/2006)

EOD 10— SIZE (1988-2003)

NAACCR Item #: 780
SAS Variable Name: EOD_10_size_1988_2003
Research: Yes
Research Limited-Field: No
Research Plus Limited-Field: No

Field Description: This item is part of the 10-digit EOD (Item #779). It records the largest dimension of the primary tumor in millimeters. Unknown size=999.

This field is only coded for cases diagnosed 1988-2003. See *SEER Extent of Disease, 1988: Codes and Coding Instructions*, Third Edition for site-specific codes and coding rules, at http://seer.cancer.gov/manuals/EOD10Dig.pub.pdf.

Note: For 2004-2015, similar type of information was collected in CS Tumor Size (2004-2015) in the collaborative stage variables.

EOD 10—EXTENT (1988-2003)

NAACCR Item #: 790
SAS Variable Name: EOD_10_extent_1988_2003
Research: Yes
Research Limited-Field: No
Research Plus Limited-Field: No

Field Description: This item codes the farthest documented extension of tumor away from the primary site, either by contiguous extension or distant metastases. Allowable values = 00-99.

This field is only coded for cases diagnosed 1988-2003. See *SEER Extent of Disease, 1988: Codes and Coding Instructions*, Third Edition for site-specific codes and coding rules, at http://seer.cancer.gov/manuals/EOD10Dig.pub.pdf.

Note: For 2004-2015, similar type of information was collected in CS extension (2004-2015) and CS Mets at DX (2004-2015) in the collaborative stage variables.

EOD 10—PROSTATE PATH EXT (1995-2003)

NAACCR Item #: 800
SAS Variable Name: EOD10Prostatepathext_1995_2003
Research: Yes
Research Limited-Field: No
Research Plus Limited-Field: No

Field Description: This is an additional field for prostate cancer only to reflect information from radical prostatectomy, effective with 1995 diagnoses. The field is left blank for all other primaries. Allowable values = 00-99.

(Continued on next page)

(Continued from EOD 10—PROSTATE PATH EXT (1995-2003))

This field is only coded for prostate cases diagnosed 1995-2003. See *SEER Extent of Disease, 1988: Codes and Coding Instructions*, Third Edition for site-specific codes and coding rules, at http://seer.cancer.gov/manuals/EOD10Dig.pub.pdf.

Note: For 2004+, similar type of information was collected in CS SSF 3 in the collaborative stage variables.

EOD 10—NODES (1995-2003)

NAACCR Item #: 810
SAS Variable Name: EOD_10_nodes_1988_2003
Research: Yes
Research Limited-Field: No
Research Plus Limited-Field: No

Field Description: This item records the highest specific lymph node chain that is involved by the tumor. Allowable values = 0-9.

This field is only coded for cases diagnosed 1988-2003. See *SEER Extent of Disease, 1988: Codes and Coding Instructions*, Third Edition for site-specific codes and coding rules, at http://seer.cancer.gov/manuals/EOD10Dig.pub.pdf.

Note: For 2004-2015, similar type of information was collected in CS Lymph Nodes (2004-2015) in the collaborative stage variables.

REGIONAL NODES POSITIVE (1988+)

NAACCR Item #: 820
SAS Variable Name: Regional_nodes_positive_1988
Research: Yes
Research Limited-Field: No
Research Plus Limited-Field: No

Field Description: Records the exact number of regional lymph nodes examined by the pathologist that were found to contain metastases.

Code	Description
00	All nodes examined are negative
01-89	Exact number of nodes positive
90	90 or more nodes are positive
95	Positive aspiration of lymph node(s) was performed
97	Positive nodes are documented, but number is unspecified
98	No nodes were examined
99	Unknown whether nodes are positive; not applicable; not stated in patient record
126	Blank

REGIONAL NODES EXAMINED (1988+)

NAACCR Item #: 830
SAS Variable Name: Regional_nodes_examined_1988
Research: Yes
Research Limited-Field: No
Research Plus Limited-Field: No

Field Description: Records the total number of regional lymph nodes that were removed and examined by the pathologist.

Code	Description
00	No nodes were examined
01-89	Exact number of nodes examined
90	90 or more nodes were examined
95	No regional nodes were removed, but aspiration of regional nodes was performed
96	Regional lymph node removal was documented as a sampling, and the number of nodes is unknown/not stated
97	Regional lymph node removal was documented as a dissection, and the number of nodes is unknown/not stated
98	Regional lymph nodes were surgically removed, but the number of lymph nodes is unknown/not stated and not documented as a sampling or dissection; nodes were examined, but the number is unknown
99	Unknown whether nodes were examined; not applicable or negative; not stated in patient record
126	Blank

EXPANDED EOD (1) – EXPANDED EOD (13)

NAACCR Item #: 840
SAS Variable Name: Expanded_EOD_1_CP65_1973_1982 --
Expanded_EOD_13_CP65_1973_1982
Research: Yes
Research Limited-Field: No
Research Plus Limited-Field: No

Field Description: Detailed site-specific codes for EOD used by SEER for selected sites of cancer for tumors diagnosed 1975-1982, except death-certificate-only cases.

For details, reference the following website:
http://seer.cancer.gov/manuals/historic/EOD_1977.pdf

2-DIGIT NS EOD / 2-DIGIT SS EOD1973-1982

NAACCR Item #: 850
SAS Variable Name: V2DigitNS_EOD_part_1_1973_1982
V2DigitNS_EOD_part_2_1973_1982
V2DigitSS_EOD_part_1_1973_1982
V2DigitSS_EOD_part_2_1973_1982
Research: Yes
Research Limited-Field: No
Research Plus Limited-Field: No

Field Description: Site-specific codes for EOD used by SEER for tumors diagnosed from January 1, 1973, to December 31, 1982, for cancer sites that did not have a 13-digit scheme. For details, reference the following website: http://seer.cancer.gov/manuals/historic/EOD_1977.pdf

---

EOD 4 – SIZE (1983-1987)
EOD 4 – EXTENT (1983-1987)
EOD 4 – NODES (1983-1987)

NAACCR Item #: 860
SAS Variable Name: EOD_4_extent_1983_1987
EOD_4_nodes_1983_1987
EOD_4_size_1983_1987
Research: Yes
Research Limited-Field: No
Research Plus Limited-Field: No

Field Description Codes for site-specific EOD used by SEER for tumors diagnosed from January 1, 1983 to December 31, 1987 for all cancer sites. For details, reference the following website: http://seer.cancer.gov/manuals/historic/EOD_1984.pdf

First and second digit- EOD 4 – SIZE (1983-1987)
Third digit- EOD 4 – EXTENT (1983-1987)
Fourth digit- EOD 4 – NODES (1983-1987)

CODING SYSTEM – EOD (1973-2003)

NAACCR Item#: 870
SAS Variable Name: Coding_system_EOD_1973_2003
Research: Yes
Research Limited-Field: No
Research Plus Limited-Field: No

Field Description: Indicates the type of SEER EOD code applied to the tumor.

Code	Description
0	2-Digit Nonspecific Extent of Disease (1973-82)
1	2-Digit Site-Specific Extent of Disease (1973-82)
2	13-Digit (expanded) Site Specific Extent of Disease (1973-82)
3	4-Digit Extent of Disease (1983-87)
4	10-Digit Extent of Disease, 1988 (1988-2003)
6	Blank

TUMOR MARKER 1 (1990-2003)

NAACCR Item #: 1150
SAS Variable Name: Tumor_marker_1_1990_2003
Research: Yes
Research Limited-Field: No
Research Plus Limited-Field: No

Field Description: This data item records prognostic indicators for breast cases (ERA 1990-2003), prostate cases (PAP 1998-2003) and testis cases (AFP 1998-2003).

Code	Description
0	None Done
1	Positive
2	Negative
3	Borderline; undetermined whether positive or negative
8	Ordered, but results not in chart
9	Unknown or no information
14	Blank

(Continued on next page)

(Continued from TUMOR MARKER 1 (1990-2003))

For Testicular Cancer Cases (AFP 1998-2003)

Code	Description
0	None Done (SX)
2	Within normal limits (S0)
4	Range 1 (S1) <1,000 ng/ml
5	Range 2 (S2) 1,000 – 10,000 ng/ml
6	Range 3 (S3) >10,000 ng/ml
8	Ordered, but results not in chart
9	Unknown or no information
14	Blank

For All Other Cases

Code	Description
9	Not applicable

All sites except Breast diagnosed 1990-1997 are coded 9.

All diagnoses before January 1, 1990 are coded 9.

All diagnoses after 2003 are blank.

For Breast and Testis cases diagnosed 2004+, this information is collected using CS Site-Specific Factor 1 (NAACCR Item #2880). For Prostate cases diagnosed 2004+, information is no longer collected.

For breast cancer cases, ERA over time is available in ER Status Recode Breast Cancer (1990+).

TUMOR MARKER 2 (1990-2003)

NAACCR Item #: 1160
SAS Variable Name: Tumor_marker_2_1990_2003
Research: Yes
Research Limited-Field: No
Research Plus Limited-Field: No

Field Description: This data item records prognostic indicators for breast cases (PRA 1990-2003) and testis cases (hCG 1998-2003).

For Breast Cancer Cases (PRA 1990-2003)

Code	Description
0	None Done
1	Positive
2	Negative
3	Borderline; undetermined whether positive or negative
8	Ordered, but results not in chart
9	Unknown or no information
14	Blank

For Testicular Cancer Cases (hCG 1998-2003)

Code	Description
0	None Done (SX)
2	Within normal limits (S0)
4	Range 1 (S1) <5,000 mlU/ml
5	Range 2 (S2) 5,000 – 50,000 mlU/ml
6	Range 3 (S3) to >50,000 mlU/ml
8	Ordered, but results not in chart
9	Unknown or no information
14	Blank

For All Other Cases

Code	Description
9	Not applicable

All sites except Breast diagnosed 1990-1997 are coded 9.

All diagnoses before January 1, 1990 are coded 9.

All diagnoses after 2003 are blank.

For Breast and Testis cases diagnosed 2004+, this information is collected using CS Site-Specific Factor 2 (NAACCR Item #2890).

For breast cancer cases, PRA over time is available in PR Status Recode Breast Cancer (1990+).

TUMOR MARKER 3 (1998-2003)

NAACCR Item #: 1170
SAS Variable Name: Tumor_marker_3_1990_2003
Research: Yes
Research Limited-Field: No
Research Plus Limited-Field: No

Field Description: This data item records prognostic indicators for testis cases (LDH 1998-2003)

Code	Description
0	None Done (SX)
2	Within normal limits (S0)
4	Range 1 (S1) <1.5 x upper limit of normal for LDH assay
5	Range 2 (S2) 1.5 – 10 x upper limit of normal for LDH assay
6	Range 3 (S3) >10 x upper limit of normal for LDH assay
8	Ordered, results not in chart
9	Unknown or no information
14	Blank

All sites except testis diagnosed 1998-2003 are coded 9.

All diagnoses before 1998 are coded 9.

All diagnoses after 2003 are blank.

For Testis cases diagnosed 2004+, this information is collected using CS Site-Specific Factor 3 (NAACCR Item # 2900).

CS TUMOR SIZE (2004-2015)

NAACCR Item #: 2800
SAS Variable Name: CS_tumor_size_2004_2015
Research: Yes
Research Limited-Field: No
Research Plus Limited-Field: No

Field Description: Information on tumor size. Available for 2004-2015 diagnosis years. Earlier cases may be converted and new codes added which weren't available for use prior to the current version of CS. For more information, see http://seer.cancer.gov/seerstat/variables/seer/ajcc-stage.

Code	Description
000	Indicates no mass or no tumor found; for example, when a tumor of a stated primary site is not found, but the tumor has metastasized.
001-988	Exact size in millimeters
989	989 millimeters or larger
990	Microscopic focus or foci only; no size of focus is given
991	Described as less than 1 cm
992	Described as less than 2 cm
993	Described as less than 3 cm
994	Described as less than 4 cm
995	Described as less than 5 cm
996-998	Site-specific codes where needed
999	Unknown; size not stated; not stated in patient record
888	Not applicable
1022	Blank

Examples:

Mammogram shows 2.5 cm breast malignancy	Code as 025 (2.5 cm = 25 millimeters)
CT of chest shows 4 cm mass in RUL	Code as 040 (4 cm = 40 mm)
Thyroidectomy specimen yields 8 mm carcinoma	Code as 008
Prostate needle biopsy shows 0.6 mm carcinoma	Code as 001 (round up six-tenths of mm)

CS EXTENSION (2004-2015)

NAACCR Item #: 2810
SAS Variable Name: CS_extension_2004_2015
Research: Yes
Research Limited-Field: No
Research Plus Limited-Field: No

Field Description: Information on extension of the tumor. Available for 2004-2015 diagnosis years. Earlier cases may be converted and new codes added which weren't available for use prior to the current version of CS. For more information, see http://seer.cancer.gov/seerstat/variables/seer/ajcc-stage.

Note: This item was originally a 2-digit field and was expanded to 3 digits during conversion. Generally, a zero was added to the right of the existing 2-digit field, except for 99 which became 999.

CS LYMPH NODES (2004-2015)

NAACCR Item #: 2830
SAS Variable Name: CS_lymph_nodes_2004_2015
Research: Yes
Research Limited-Field: No
Research Plus Limited-Field: No

Field Description: Information on involvement of lymph nodes. Available for 2004-2015 diagnosis years. Earlier cases may be converted and new codes added which weren't available for use prior to the current version of CS. For more information, see http://seer.cancer.gov/seerstat/variables/seer/ajcc-stage.

Note: This item was originally a 2-digit field and was expanded to 3 digits during conversion. Generally, a zero was added to the right of the existing 2-digit field, except for 99 which became 999.

CS METS AT DX (2004-2015)

NAACCR Item #: 2850
SAS Variable Name: CS_mets_at_dx_2004_2015
Research: Yes
Research Limited-Field: No
Research Plus Limited-Field: No

Field Description: Information on distant metastasis. Available for 2004-2015 diagnosis years. Earlier cases may be converted and new codes added which weren't available for use prior to the current version of CS. For more information, see http://seer.cancer.gov/seerstat/variables/seer/ajcc-stage.

CS SITE-SPECIFIC FACTOR 1 (2004-2017 varying by schema)

NAACCR Item #: 2880
SAS Variable Name: CSsitespecificfactor120042017va
Research: Yes
Research Limited-Field: No
Research Plus Limited-Field: No

Field Description: Each CS site-specific factor (SSF) is schema dependent. They can provide information needed to stage the case, clinically relevant information, or prognostic information. Available for varying years and schemas depending on standard setter requirements. Earlier cases may be converted and new codes added which weren't available for use prior to the current version of CS.

For more information, see variable documentation.

CS SITE-SPECIFIC FACTOR 2 (2004-2017 varying by schema)

NAACCR Item #: 2890
SAS Variable Name: CSsitespecificfactor220042017va
Research: Yes
Research Limited-Field: No
Research Plus Limited-Field: No

Field Description: Each CS site-specific factor (SSF) is schema dependent. They can provide information needed to stage the case, clinically relevant information, or prognostic information. Available for varying years and schemas depending on standard setter requirements. Earlier cases may be converted and new codes added which weren't available for use prior to the current version of CS.

For more information, see variable documentation.

CS SITE-SPECIFIC FACTOR 3 (2004-2017 varying by schema)

NAACCR Item #: 2900
SAS Variable Name: CSsitespecificfactor320042017va
Research: Yes
Research Limited-Field: No
Research Plus Limited-Field: No

Field Description: Each CS site-specific factor (SSF) is schema dependent. They can provide information needed to stage the case, clinically relevant information, or prognostic information. Available for varying years and schemas depending on standard setter requirements. Earlier cases may be converted and new codes added which weren't available for use prior to the current version of CS.

For more information, see variable documentation.
.

CS SITE-SPECIFIC FACTOR 4 (2004-2017 varying by schema)

NAACCR Item #: 2910
SAS Variable Name: CSsitespecificfactor420042017va
Research: Yes
Research Limited-Field: No
Research Plus Limited-Field: No

Field Description: Each CS site-specific factor (SSF) is schema dependent. They can provide information needed to stage the case, clinically relevant information, or prognostic information. Available for varying years and schemas depending on standard setter requirements. Earlier cases may be converted and new codes added which weren't available for use prior to the current version of CS.

For more information, see variable documentation.

CS SITE-SPECIFIC FACTOR 5 (2004-2017 varying by schema)

NAACCR Item #: 2920
SAS Variable Name: CSsitespecificfactor520042017va
Research: Yes
Research Limited-Field: No
Research Plus Limited-Field: No

Field Description: Each CS site-specific factor (SSF) is schema dependent. They can provide information needed to stage the case, clinically relevant information, or prognostic information. Available for varying years and schemas depending on standard setter requirements. Earlier cases may be converted and new codes added which weren't available for use prior to the current version of CS.

For more information, see variable documentation.

CS SITE-SPECIFIC FACTOR 6 (2004-2017 varying by schema)

NAACCR Item #: 2930
SAS Variable Name: CSsitespecificfactor620042017va
Research: Yes
Research Limited-Field: No
Research Plus Limited-Field: No

Field Description: Each CS site-specific factor (SSF) is schema dependent. They can provide information needed to stage the case, clinically relevant information, or prognostic information. Available for varying years and schemas depending on standard setter requirements. Earlier cases may be converted and new codes added which weren't available for use prior to the current version of CS.

For more information, see variable documentation.

CS SITE-SPECIFIC FACTOR 7 (2004-2017 varying by schema)

NAACCR Item #: 2861
SAS Variable Name: CSsitespecificfactor720042017va
Research: Yes
Research Limited-Field: No
Research Plus Limited-Field: No

Field Description: Each CS site-specific factor (SSF) is schema dependent. They can provide information needed to stage the case, clinically relevant information, or prognostic information. Available for varying years and schemas depending on standard setter requirements. Earlier cases may be converted and new codes added which weren't available for use prior to the current version of CS.

For more information, see variable documentation.

CS SITE-SPECIFIC FACTOR 8 (2004-2017 varying by schema)

NAACCR Item #: 2862
SAS Variable Name: CSsitespecificfactor820042017va
Research: Yes
Research Limited-Field: No
Research Plus Limited-Field: No

Field Description: Each CS site-specific factor (SSF) is schema dependent. They can provide information needed to stage the case, clinically relevant information, or prognostic information. Available for varying years and schemas depending on standard setter requirements. Earlier cases may be converted and new codes added which weren't available for use prior to the current version of CS.

For more information, see variable documentation.

CS SITE-SPECIFIC FACTOR 9 (2004-2017 varying by schema)

NAACCR Item #: 2863
SAS Variable Name: CSsitespecificfactor920042017va
Research: Yes
Research Limited-Field: No
Research Plus Limited-Field: No

Field Description: Each CS site-specific factor (SSF) is schema dependent. They can provide information needed to stage the case, clinically relevant information, or prognostic information. Available for varying years and schemas depending on standard setter requirements. Earlier cases may be converted and new codes added which weren't available for use prior to the current version of CS.

For more information, see variable documentation.

CS SITE-SPECIFIC FACTOR 10 (2004-2017 varying by schema)

NAACCR Item #: 2864
SAS Variable Name: CSsitespecificfactor1020042017v
Research: Yes
Research Limited-Field: No
Research Plus Limited-Field: No

Field Description: Each CS site-specific factor (SSF) is schema dependent. They can provide information needed to stage the case, clinically relevant information, or prognostic information. Available for varying years and schemas depending on standard setter requirements. Earlier cases may be converted and new codes added which weren't available for use prior to the current version of CS.

For more information, see variable documentation.

CS SITE-SPECIFIC FACTOR 11 (2004-2017 varying by schema)

NAACCR Item #: 2865
SAS Variable Name: CSsitespecificfactor1120042017v
Research: Yes
Research Limited-Field: No
Research Plus Limited-Field: No

Field Description: Each CS site-specific factor (SSF) is schema dependent. They can provide information needed to stage the case, clinically relevant information, or prognostic information. Available for varying years and schemas depending on standard setter requirements. Earlier cases may be converted and new codes added which weren't available for use prior to the current version of CS.

For more information, see variable documentation.

CS SITE-SPECIFIC FACTOR 12 (2004-2017 varying by schema)

NAACCR Item #: 2866
SAS Variable Name: CSsitespecificfactor1220042017v
Research: Yes
Research Limited-Field: No
Research Plus Limited-Field: No

Field Description: Each CS site-specific factor (SSF) is schema dependent. They can provide information needed to stage the case, clinically relevant information, or prognostic information. Available for varying years and schemas depending on standard setter requirements. Earlier cases may be converted and new codes added which weren't available for use prior to the current version of CS.

For more information, see variable documentation.

CS SITE-SPECIFIC FACTOR 13 (2004-2017 varying by schema)

NAACCR Item #: 2867
SAS Variable Name: CSsitespecificfactor1320042017v
Research: Yes
Research Limited-Field: No
Research Plus Limited-Field: No

Field Description: Each CS site-specific factor (SSF) is schema dependent. They can provide information needed to stage the case, clinically relevant information, or prognostic information. Available for varying years and schemas depending on standard setter requirements. Earlier cases may be converted and new codes added which weren't available for use prior to the current version of CS.

For more information, see variable documentation.

CS SITE-SPECIFIC FACTOR 15 (2004-2017 varying by schema)

NAACCR Item #: 2869
SAS Variable Name: CSsitespecificfactor1520042017v
Research: Yes
Research Limited-Field: No
Research Plus Limited-Field: No

Field Description: Each CS site-specific factor (SSF) is schema dependent. They can provide information needed to stage the case, clinically relevant information, or prognostic information. Available for varying years and schemas depending on standard setter requirements. Earlier cases may be converted and new codes added which weren't available for use prior to the current version of CS.

For more information, see variable documentation.

CS SITE-SPECIFIC FACTOR 16 (2004-2017 varying by schema)

NAACCR Item #: 2870
SAS Variable Name: CSsitespecificfactor1620042017v
Research: Yes
Research Limited-Field: No
Research Plus Limited-Field: No

Field Description: Each CS site-specific factor (SSF) is schema dependent. They can provide information needed to stage the case, clinically relevant information, or prognostic information. Available for varying years and schemas depending on standard setter requirements. Earlier cases may be converted and new codes added which weren't available for use prior to the current version of CS.

For more information, see variable documentation.

CS SITE-SPECIFIC FACTOR 25 (2004-2017 varying by schema)

NAACCR Item #: 2879
SAS Variable Name: CSsitespecificfactor2520042017v
Research: Yes
Research Limited-Field: No
Research Plus Limited-Field: No

Field Description: Each CS site-specific factor (SSF) is schema dependent. They can provide information needed to stage the case, clinically relevant information, or prognostic information. Available for varying years and schemas depending on standard setter requirements. Earlier cases may be converted and new codes added which weren't available for use prior to the current version of CS.

For more information, see variable documentation.

DERIVED AJCC T, 6th ED (2004-2015)

NAACCR Item #: 2940
SAS Variable Name: DerivedAJCC_T_6th_ed_2004_2015
Research: Yes
Research Limited-Field: No
Research Plus Limited-Field: No

Field Description: This is the AJCC "T" component that is derived from CS coded fields, using the CS algorithm, effective with 2004-2015 diagnosis years. For more information, see variable documentation.

Code	Description
99	TX
00	T0
01	Ta
05	Tis
06	Tispu (Urethra only)
07	Tispd (Urethra only)
10	T1
11	T1mic
19	T1 NOS
12	T1a
13	T1a1
14	T1a2
15	T1b
16	T1b1
17	T1b2
18	T1c
20	T2
29	T2 NOS
21	T2a
22	T2b
23	T2c
30	T3
39	T3 NOS
31	T3a
32	T3b
33	T3c
40	T4
49	T4 NOS
41	T4a
42	T4b
43	T4c
44	T4d
80	T1a NOS
81	T1b NOS
88	Not applicable
126	Blank

DERIVED AJCC N, 6th ED (2004-2015)

NAACCR Item #: 2960
SAS Variable Name: DerivedAJCC_N_6th_ed_2004_2015
Research: Yes
Research Limited-Field: No
Research Plus Limited-Field: No

Field Description: This is the AJCC "N" component that is derived from CS coded fields, using the CS algorithm, effective with 2004-2015 diagnosis years. For more information, see variable documentation.

Code	Description
99	NX
00	N0
01	N0(i-)
02	N0(i+)
03	N0(mol-)
04	N0(mol+)
10	N1
19	N1 NOS
11	N1a
12	N1b
13	N1c
18	N1mi
20	N2
29	N2 NOS
21	N2a
22	N2b
23	N2c
30	N3
39	N3 NOS
31	N3a
32	N3b
33	N3c
88	Not applicable
126	Blank

DERIVED AJCC M, 6th ED (2004-2015)

NAACCR Item #: 2980
SAS Variable Name: DerivedAJCC_M_6th_ed_2004_2015
Research: Yes
Research Limited-Field: No
Research Plus Limited-Field: No

Field Description: This is the AJCC "M" component that is derived from CS coded fields, using the CS algorithm, effective with 2004-2015 diagnosis years. For more information, see variable documentation.

Code	Description
99	MX
00	M0
10	M1
11	M1a
12	M1b
13	M1c
19	M1 NOS
88	Not applicable
126	Blank

DERIVED AJCC STAGE GROUP, 6th ED (2004-2015)

NAACCR Item #: 3000
SAS Variable Name: DerivedAJCCStageGroup6thed2004
Research: Yes
Research Limited-Field: No
Research Plus Limited-Field: No

Field Description: This is the AJCC "Stage Group" component that is derived from CS detailed site-specific codes, using the CS algorithm, effective with 2004-2015 diagnosis years. For more information, see variable documentation.

Code	Description
00	Stage 0
01	Stage 0a
02	Stage 0is
10	Stage I
11	Stage I NOS
12	Stage IA
13	Stage IA1
14	Stage IA2
15	Stage IB
16	Stage IB1
17	Stage IB2
18	Stage IC

(Continued on next page)

(Continued from DERIVED AJCC STAGE GROUP, 6th ED (2004-2015))

Code	Description
19	Stage IS
23	Stage ISA (lymphoma only)
24	Stage ISB (lymphoma only)
20	Stage IEA (lymphoma only)
21	Stage IEB (lymphoma only)
22	Stage IE (lymphoma only)
30	Stage II
31	Stage II NOS
32	Stage IIA
33	Stage IIB
34	Stage IIC
35	Stage IIEA (lymphoma only)
36	Stage IIEB (lymphoma only)
37	Stage IIE (lymphoma only)
38	Stage IISA (lymphoma only)
39	Stage IISB (lymphoma only)
40	Stage IIS (lymphoma only)
41	Stage IIESA (lymphoma only)
42	Stage IIESB (lymphoma only)
43	Stage IIES (lymphoma only)
50	Stage III
51	Stage III NOS
52	Stage IIIA
53	Stage IIIB
54	Stage IIIC
55	Stage IIIEA (lymphoma only)
56	Stage IIIEB (lymphoma only)
57	Stage IIIE (lymphoma only)
58	Stage IIISA (lymphoma only)
59	Stage IIISB (lymphoma only)
60	Stage IIIS (lymphoma only)
61	Stage IIIESA (lymphoma only)
62	Stage IIIESB (lymphoma only)
63	Stage IIIES (lymphoma only)
70	Stage IV
71	Stage IV NOS
72	Stage IVA
73	Stage IVB
74	Stage IVC
88	Not applicable
90	Stage Occult
99	Stage Unknown
126	Blank

SUMMARY STAGE 2000 (1998-2017)

NAACCR Item #: N/A
SAS Variable Name: Summary_stage_2000_1998_2017
Research: Yes
Research Limited-Field: Yes
Research Plus Limited-Field: Yes

Field Description: Combination of NAACCR Items #759 and #3020. For 2004-2015 cases, it is copied from Derived SS2000 and for 2016+, it is the best available between Derived SS2000 and SEER Summary Stage 2000. All benign/borderline cases are set to NA.

COMBINED SUMMARY STAGE (2004+)

NAACCR Item #: N/A
SAS Variable Name: Combined_Summary_Stage_2004
Research: Yes
Research Limited-Field: No
Research Plus Limited-Field: Yes

Field Description: Combined Summary Stage field to facilitate stage analyses over time. Created from SEER Combined Summary Stage 2000 (2004-2017) & Derived Summary Stage 2018 (2018+). For more information including sites, years and registries for which it isn't calculated, see https://seer.cancer.gov/seerstat/variables/seer/lrd-stage/

CS VERSION INPUT ORIGINAL (2004-2015)

NAACCR Item #: 2935
SAS Variable Name: CSversioninputoriginal20042015
Research: Yes
Research Limited-Field: No
Research Plus Limited-Field: No

Field Description: This item indicates the number of the version used to initially code CS fields. The first two digits represent the major version number; the second two digits represent minor version changes; and, the last two digits represent even less significant changes, such as corrections of typographical errors that do not affect coding or derivation of results.

This data item along with CS version input current gives info on what document to use for the CS codes. For more information, see http://seer.cancer.gov/seerstat/variables/seer/ajcc-stage.

CS VERSION DERIVED (2004-2015)

NAACCR Item #: 2936
SAS Variable Name: CS_version_derived_2004_2015
Research: Yes
Research Limited-Field: No
Research Plus Limited-Field: No

Field Description: This item indicates the number of the version of the CS used most recently to derive the CS output fields. The first two digits represent the major version number; the second two digits represent minor version changes; and, the last two digits represent even less significant changes, such as corrections of typographical errors that do not affect coding or derivation of results.

CS VERSION INPUT CURRENT (2004-2015)

NAACCR Item #: 2937
SAS Variable Name: CSversioninputcurrent2004_2015
Research: Yes
Research Limited-Field: No
Research Plus Limited-Field: No

Field Description: This item indicates the number of the version of the CS after input fields have been updated or recoded. The first two digits represent the major version number; the second two digits represent minor version changes; and, the last two digits represent even less significant changes, such as corrections of typographical errors that do not affect coding or derivation of results.

This data item along with CS version input original gives info on what document to use for the CS codes. For more information, see http://seer.cancer.gov/seerstat/variables/seer/ajcc-stage.

RX SUMM—SURG PRIM SITE (1998+)

NAACCR Item #: 1290
SAS Variable Name: RX_Summ_Surg_Prim_Site_1998
Research: Yes
Research Limited-Field: No
Research Plus Limited-Field: No

Field Description: Surgery of Primary Site describes a surgical procedure that removes and/or destroys tissue of the primary site performed as part of the initial work-up or first course of therapy. The actual coding schemes for individual sites and diagnosis years can be viewed here

General Coding Structure

Code	Description
00	None; no surgical procedure of primary site; diagnosed at autopsy only
10-19	Site-specific codes. Tumor destruction; no pathologic specimen or unknown whether there is a pathologic specimen
20-80	Site-specific codes. Resection; pathologic specimen
90	Surgery, NOS. A surgical procedure to the primary site was done, but no information on the type of surgical procedure is provided.
98	Special codes for hematopoietic, reticuloendothelial, immunoproliferative, myeloproliferative diseases; ill-defined sites; and unknown primaries (See site-specific codes for the sites and histologies), except death certificate only
99	Unknown if surgery performed; death certificate only
126	Blank

For diagnosis years 1998-2002, SEER registries were required to convert field RX Summ—Surg Site 98-02 (NAACCR Item #1646) to RX Summ—Surg Prim Site.

RX SUMM—SCOPE REG LN SUR (2003+)

NAACCR Item #: 1292
SAS Variable Name: RX_Summ_Scope_Reg_LN_Sur_2003
Research: Yes
Research Limited-Field: No
Research Plus Limited-Field: No

Field Description: Scope of Regional Lymph Node Surgery describes the procedure of removal, biopsy, or aspiration of **regional** lymph nodes performed during the initial work-up or first course of therapy at all facilities.

Changed to Unknown or Not Applicable for breast cancer cases. See http://seer.cancer.gov/seerstat/variables/seer/regional_ln/ for more information.

Code	Description
0	No regional lymph nodes removed or aspirated; diagnosed at autopsy.
1	Biopsy or aspiration of regional lymph node, NOS
2	Sentinel lymph node biopsy [only]
3	Number of regional lymph nodes removed unknown, not stated; regional lymph nodes removed, NOS
4	1 to 3 regional lymph nodes removed
5	4 or more regional lymph nodes removed
6	Sentinel node biopsy and code 3, 4, or 5 at same time or timing not noted
7	Sentinel node biopsy and code 3, 4, or 5 at different times
9	Unknown or not applicable; death certificate only
14	Blank

RX SUMM-SURG OTH REG/DIS (2003+)

NAACCR Item #: 1294
SAS Variable Name: RX_Summ_Surg_Oth_Reg_Dis_2003
Research: Yes
Research Limited-Field: No
Research Plus Limited-Field: No

Field Description: Surgical procedure of Other Site describes the surgical removal of distant lymph node(s) or other tissue(s) or organ(s) beyond the primary site.

Code	Description
0	None; diagnosed at autopsy
1	Nonprimary surgical procedure performed
2	Nonprimary surgical procedure to other regional sites
3	Non-primary surgical procedure to distant lymph node(s)
4	Nonprimary surgical procedure to distant site
5	Combination of codes 2, 3, or 4
9	Unknown; death certificate only
14	Blank

RX SUMM-REG LN EXAMINED (1998-2002)

NAACCR Item #: 1296
SAS Variable Name: RXSummRegLN_Examined_1998_2002
Research: Yes
Research Limited-Field: No
Research Plus Limited-Field: No

Field Description: This data item records the number of regional lymph nodes examined in conjunction with surgery performed as part of the first course of treatment at all facilities. This item is only available for cases diagnosed 1998-2002.

Code	Description
00	No regional lymph nodes examined
01	One regional lymph node examined
02	Two regional lymph nodes examined
..	(Actual number of lymph nodes examined)
90	90 or more regional lymph nodes examined
95	No regional lymph nodes examined
96	Regional lymph node removal documented as sampling, and number of lymph nodes unknown/not stated
97	Regional lymph node removal documented as dissection, and number of lymph nodes unknown/not stated
98	Regional lymph nodes surgically removed, but number of lymph nodes unknown/not stated and not documented as sampling or dissection
99	Unknown; not stated; death certificate-only
126	Blank

RX SUMM-SYSTEMIC SUR SEQ

NAACCR Item #: 1639
SAS Variable Name: RX_Summ_Systemic_Surg_Seq
Research: No
Research Limited-Field: No
Research Plus Limited-Field: No

Field Description: This data item records the sequencing of systemic therapy and surgical procedures given as part of first course of treatment.

Code	Description
0	No systemic therapy and/or surgical procedures; unknown if surgery and/or systemic therapy given
2	Systemic therapy before surgery
3	Systemic therapy after surgery
4	Systemic therapy both before and after surgery
5	Intraoperative systemic therapy
6	Intraoperative systemic therapy with other therapy administered before and/or after surgery
7	Surgery both before and after systemic therapy
9	Sequence unknown, but both surgery and systemic therapy given

RX SUMM--SURG/RAD SEQ

NAACCR Item #: 1380
SAS Variable Name: Radiation_sequence_with_surgery
Research: No
Research Limited-Field: No
Research Plus Limited-Field: No

Field Description: This field records the order in which surgery and radiation therapies were administered for those patients who had both surgery and radiation.

Code	Description
0	No radiation and/or surgery as defined above
2	Radiation before surgery
3	Radiation after surgery
4	Radiation both before and after surgery
5	Intraoperative radiation
6	Intraoperative radiation with other radiation given before and/or after surgery
7	Surgery both before and after radiation
9	Sequence unknown, but both surgery and radiation were given

REASON NO CANCER-DIRECTED SURGERY

NAACCR Item #: 1340
SAS Variable Name: Reasonnocancerdirected_surgery
Research: Yes
Research Limited-Field: No
Research Plus Limited-Field: No

Field Description: This data item documents the reason that surgery was not performed.

Code	Description
0	Surgery performed

No surgery

Code	Description
1*	Surgery not recommended
2*	Contraindicated due to other conditions; Autopsy Only case (1973-2002)
5	Patient died before recommended surgery
6	Unknown reason for no surgery
7*	Patient or patient's guardian refused

Unknown if surgery performed

Code	Description
8	Recommended, unknown if done
9	Unknown if surgery performed; Death Certificate Only case; Autopsy only case (2003+)
14	Blank

*Codes not used prior to 1988. Code '2' used only for Autopsy Only cases prior to 1988.

RADIATION RECODE

NAACCR Item #: N/A
SAS Variable Name: Radiation_recode
Research: No
Research Limited-Field: No
Research Plus Limited-Field: No

Field Description: This data item indicates the method of radiation therapy performed as part of the first course of treatment. It is a recode of NAACCR Item #1360.

Code	Description
0	None/Unknown; diagnosed at autopsy
1	Beam radiation
2	Radioactive implants
3	Radioisotopes
4	Combination of 1 with 2 or 3
5	Radiation, NOS—method or source not specified
6	Other radiation (1973-1987 cases only)
7	Patient or patient's guardian refused radiation therapy
8	Radiation recommended, unknown if administered

CHEMOTHERAPY RECODE (YES, NO/UNK)

NAACCR Item #: N/A
SAS Variable Name: Chemotherapy_recode_yes_no_unk
Research: No
Research Limited-Field: No
Research Plus Limited-Field: No

Field Description: This field records whether chemotherapy was given

Code	Description
0	None/Unknown
1	Yes

SITE SPECIFIC SURGERY (1973-1997 VARYING DETAIL BY YEAR AND SITE)

NAACCR Item #: 1640
SAS Variable Name: Sitespecificsurgery19731997var
Research: Yes
Research Limited-Field: No
Research Plus Limited-Field: No

Field Description: For detailed information on individual sites and time periods please see: http://seer.cancer.gov/seerstat/variables/seer/surgery/

This field specifies information on surgery during first course of therapy whether it was cancer-directed or not. (Prior to 1988 SEER did not collect information on surgical procedures if not cancer-directed.) The Reason for No Cancer-directed Surgery field must be used to distinguish among no cancer-directed surgery performed; cancer-directed surgery recommended, unknown if performed; and unknown if cancer-directed surgery performed.

SCOPE OF REG LYMPH ND SURG (1998-2002)

NAACCR Item #: 1647
SAS Variable Name: Scopeofreglymphndsurg1998_2002
Research: Yes
Research Limited-Field: No
Research Plus Limited-Field: No

Field Description: This field describes the removal, biopsy or aspiration of regional lymph node(s) at the time of surgery of the primary site or during a separate surgical event at all facilities for cases diagnosed 1998-2002.

Changed to Unknown or Not Applicable for breast cancer cases. See http://seer.cancer.gov/seerstat/variables/seer/regional_ln/ for more information.

See http://seer.cancer.gov/manuals/historic/AppendC.pdf for a list of valid codes.

SURGERY OF OTH REG/DIS SITES (1998-2002)

NAACCR Item #: 1648
SAS Variable Name: Surgeryofothregdissites1998200
Research: Yes
Research Limited-Field: No
Research Plus Limited-Field: No

Field Description: This field records the removal of distant lymph nodes or other tissue(s)/organ(s) beyond the primary site given at all facilities as part of the first course of treatment for cases diagnosed 1998-2002.

See http://seer.cancer.gov/manuals/historic/AppendC.pdf for a list of valid codes.

RECORD NUMBER RECODE

NAACCR Item #: 1775
SAS Variable Name: Record_number_recode
Research: Yes
Research Limited-Field: Yes
Research Plus Limited-Field: Yes

Field Description: This variable sequentially numbers a person's tumors. The ordered values are based on date of diagnosis and then sequence number. This variable is used as part of the algorithm for calculating the survival time recode variables (NAACCR items 1782-1788) and is used for survival, prevalence, and multiple primary – standardized incidence ratio analyses in SEER*Stat.

Code	Description
01	Record number 01 for patient
02	Record number 02 for patient
..	..
..	..
99	Record number 99 for patient

AGE RECODE WITH <1 YEAR OLDS

NAACCR Item #: N/A
SAS Variable Name: Age_recode_with_1_year_olds
Research: Yes
Research Limited-Field: Yes
Research Plus Limited-Field: Yes

Field Description: The age recode variable is based on Age at Diagnosis (single-year ages). The groupings used in the age recode variable are determined by the age groupings in the population data. This recode has 19 age groups in the age recode variable (< 1 year, 1-4 years, 5-9 years, ..., 85+ years).

Code	Description
00	Age 00
01	Ages 01-04
02	Ages 05-09
03	Ages 10-14
04	Ages 15-19
05	Ages 20-24
06	Ages 25-29
07	Ages 30-34
08	Ages 35-39
09	Ages 40-44
10	Ages 45-49
11	Ages 50-54
12	Ages 55-59
13	Ages 60-64
14	Ages 65-69
15	Ages 70-74
16	Ages 75-79
17	Ages 80-84
18	Ages 85+
29	Unknown Age

SITE RECODE ICD-O-3/WHO 2008

NAACCR Item #: N/A
SAS Variable Name: Site_recode_ICD_O_3_WHO_2008
Research: Yes
Research Limited-Field: Yes
Research Plus Limited-Field: Yes

Field Description: A recode based on Primary Site and ICD-O-3 Histology in order to make analyses of site/histology groups easier. For example, the lymphomas are excluded from stomach and Kaposi and mesothelioma are separate categories based on histology. For more information, see http://seer.cancer.gov/siterecode/icdo3_dwhoheme/index.html

---

SITE RECODE ICD-O-3/WHO 2008 (for SIRs)

NAACCR Item #: N/A
SAS Variable Name: SiterecodeICDO3WHO2008_for_SIRs
Research: Yes
Research Limited-Field: Yes
Research Plus Limited-Field: Yes

Field Description: Preferred site categorization for use with Standardized Incidence Ratios (SIRs). For more information, see https://seer.cancer.gov/siterecode

---

SITE RECODE – RARE TUMORS

NAACCR Item #: N/A
SAS Variable Name: Site_recode_rare_tumors
Research: Yes
Research Limited-Field: Yes
Research Plus Limited-Field: Yes

Field Description: Cancer type grouping to define clinically relevant, histologically defined rare cancers. For more information, see https://seer.cancer.gov/seerstat/variables/seer/raresiterecode/

BEHAVIOR RECODE FOR ANALYSIS

NAACCR Item #: N/A
SAS Variable Name: Behavior_recode_for_analysis
Research: Yes
Research Limited-Field: Yes
Research Plus Limited-Field: Yes

Field Description: This recode was created so that data analyses could eliminate major groups of histologies/behaviors that weren't collected consistently over time, for example benign brain, myelodysplastic syndromes, and borderline tumors of the ovary. Created from ICD-O-3 behavior and histology. For more information, see http://seer.cancer.gov/behavrecode.

Code	Description
0	Benign
1	Borderline malignancy
2	In situ
3	Malignant
4	Only malignant in ICD-O-3
5	No longer reportable in ICD-O-3
6	Only malignant 2010+

HISTOLOGY RECODE—BROAD GROUPINGS

NAACCR Item #: N/A
SAS Variable Name: Histologyrecodebroad_groupings
Research: Yes
Research Limited-Field: Yes
Research Plus Limited-Field: Yes

Field Description: Based on Histologic Type ICD-O-3.

Code	Description
00	8000-8009 : unspecified neoplasms
01	8010-8049 : epithelial neoplasms, NOS
02	8050-8089 : squamous cell neoplasms
03	8090-8119 : basal cell neoplasms
04	8120-8139 : transitional cell papillomas and carcinomas
05	8140-8389 : adenomas and adenocarcinomas
06	8390-8429 : adnexal and skin appendage neoplasms
07	8430-8439 : mucoepidermoid neoplasms
08	8440-8499 : cystic, mucinous and serous neoplasms
09	8500-8549 : ductal and lobular neoplasms
10	8550-8559 : acinar cell neoplasms
11	8560-8579 : complex epithelial neoplasms
12	8580-8589 : thymic epithelial neoplasms
13	8590-8679 : specialized gonadal neoplasms
14	8680-8719 : paragangliomas and glumus tumors
15	8720-8799 : nevi and melanomas
16	8800-8809 : soft tissue tumors and sarcomas, NOS
17	8810-8839 : fibromatous neoplasms
18	8840-8849 : myxomatous neoplasms
19	8850-8889 : lipomatous neoplasms
20	8890-8929 : myomatous neoplasms
21	8930-8999 : complex mixed and stromal neoplasms
22	9000-9039 : fibroepithelial neoplasms
23	9040-9049 : synovial-like neoplasms
24	9050-9059 : mesothelial neoplasms
25	9060-9099 : germ cell neoplasms
26	9100-9109 : trophoblastic neoplasms
27	9110-9119 : mesonephromas
28	9120-9169 : blood vessel tumors
29	9170-9179 : lymphatic vessel tumors

(Continued on next page)

(Continued from HISTOLOGY RECODE—BROAD GROUPINGS)

Code	Description
30	9180-9249 : osseous and chondromatous neoplasms
31	9250-9259 : giant cell tumors
32	9260-9269 : miscellaneous bone tumors (C40._,C41._)
33	9270-9349 : odontogenic tumors (C41._)
34	9350-9379 : miscellaneous tumors
35	9380-9489 : gliomas
36	9490-9529 : neuroepitheliomatous neoplasms
37	9530-9539: meningiomas
38	9540-9579 : nerve sheath tumors
39	9580-9589 : granular cell tumors & alveolar soft part sarcomas
40	9590-9599 : malignant lymphomas, NOS or diffuse
41	9650-9669 : hodgkin lymphomas
42	9670-9699 : nhl - mature b-cell lymphomas
43	9700-9719 : nhl - mature t and nk-cell lymphomas
44	9720-9729 : nhl - precursor cell lymphoblastic lymphoma
45	9730-9739 : plasma cell tumors
46	9740-9749 : mast cell tumors
47	9750-9759 : neoplasms of histiocytes and accessory lymphoid cells
48	9760-9769 : immunoproliferative diseases
49	9800-9805: leukemias, nos
50	9820-9839 : lymphoid leukemias (C42.1)
51	9840-9939 : myeloid leukemias (C42.1)
52	9940-9949 : other leukemias (C42.1)
53	9950-9969 : chronic myeloproliferative disorders (C42.1)
54	9970-9979 : other hematologic disorders
55	9980-9989 : myelodysplastic syndrome
98	other

HISTOLOGY RECODE—BRAIN GROUPINGS

NAACCR Item #: N/A
SAS Variable Name: HistologyrecodeBrain_groupings
Research: Yes
Research Limited-Field: Yes
Research Plus Limited-Field: Yes

Field Description: Based on Histologic Type ICD-O-3.

Code	Description
01	Diffuse astrocytoma (protoplasma, fibrillary)
02	Anaplastic astrocytoma
03	Glioblastoma
04	Pilocytic astrocytoma
05	Unique astrocytoma variants
06	Oligodendroglioma
07	Anaplastic oligodendroglioma
08	Ependymoma/anaplastic ependymoma
09	Ependymoma variants
10	Mixed glioma
11	Astrocytoma, NOS
12	Glioma , NOS
13	Choroid plexus
14	Neuroepithelial
15	Benign & malignant neuronal/glial, neuronal & mixed
16	Pineal parenchymal
17	Embryonal/primitive/medulloblastoma
18	Nerve sheath, benign and malignant
19	Meningioma, benign and malignant
20	Other mesenchymal, benign and malignant
21	Hemangioma and hemagioblastoma
22	Lymphoma
23	Germ cell tumors, cysts, and heterotopias
24	Chordoma/chondrosarcoma
25	Pituitary
26	Craniopharyngioma
27	Neoplasm, unspecified, benign and malignant
97	Other Brain Histologies
98	Not Brain

ICCC SITE RECODE EXTENDED 3RD EDITION/IARC 2017

NAACCR Item #: N/A
SAS Variable Name: ICCCsiterecodeextended3rdediti
Research: Yes
Research Limited-Field: Yes
Research Plus Limited-Field: Yes

Field Description: Based on ICD-O-3. For more information on this International Classification of Childhood Cancer (ICCC) site/histology recode, see https://seer.cancer.gov/iccc. While the recode is normally used for childhood cancers, it is on the file for all ages so that childhood cancers could be compared across age groups. For more information, see https://seer.cancer.gov/iccc/iccc-iarc-2017.html

---

TNM 7/CS v0204+ Schema (thru 2017)

NAACCR Item #: N/A
SAS Variable Name: TNM_7_CS_v0204_Schema_thru_2017
Research: Yes
Research Limited-Field: No
Research Plus Limited-Field: No

Field Description: Information is collected under the specifications of a particular schema based on site and histology. This recode should be used in any analysis of 7th ed stage and T, N, M.

(Table begins on next page)

(Continued from TNM 7/CS v0204+ (thru 2017))

Code	Description
001	AdnexaUterineOther
002	AdrenalGland
003	AmpullaVater
004	Anus
005	Appendix
006	BileDuctsDistal
007	BileDuctsIntraHepat
008	BileDuctsPerihilar
009	BiliaryOther
010	Bladder
011	Bone
012	Brain
013	Breast
014	BuccalMucosa
015	CarcinoidAppendix
016	Cervix
017	CNSOther
018	Colon
019	Conjunctiva
020	CorpusAdenosarcoma
021	CorpusCarcinoma
022	CorpusSarcoma
023	CysticDuct
024	DigestiveOther
025	EndocrineOther
026	EpiglottisAnterior
027	Esophagus
028	EsophagusGEJunction

(Continued on next page)

(Continued from TNM 7/CS v0204+ (thru 2017))

Code	Description
029	EyeOther
030	FallopianTube
031	FloorMouth
032	Gallbladder
033	GenitalFemaleOther
034	GenitalMaleOther
035	GISTAppendix
036	GISTColon
037	GISTEsophagus
038	GISTPeritoneum
039	GISTRectum
040	GISTSmallIntestine
041	GISTStomach
042	GumLower
043	GumOther
044	GumUpper
045	HeartMediastinum
046	HemeRetic
047	Hypopharynx
048	IllDefinedOther
049	IntracranialGland
050	KaposiSarcoma
051	KidneyParenchyma
052	KidneyRenalPelvis
053	LacrimalGland
054	LacrimalSac
055	LarynxGlottic
056	LarynxOther
057	LarynxSubglottic
058	LarynxSupraglottic
059	LipLower
060	LipOther
061	LipUpper
062	Liver
063	Lung

(Continued on next page)

(Continued from TNM 7/CS v0204+ (thru 2017))

Code	Description
064	Lymphoma
065	LymphomaOcularAdnexa
066	MelanomaBuccalMucosa
067	MelanomaChoroid
068	MelanomaCiliaryBody
069	MelanomaConjunctiva
070	MelanomaEpiglottisAnterior
071	MelanomaEyeOther
072	MelanomaFloorMouth
073	MelanomaGumLower
074	MelanomaGumOther
075	MelanomaGumUpper
076	MelanomaHypopharynx
077	MelanomaIris
078	MelanomaLarynxGlottic
079	MelanomaLarynxOther
080	MelanomaLarynxSubglottic
081	MelanomaLarynxSupraglottic
082	MelanomaLipLower
083	MelanomaLipOther
084	MelanomaLipUpper
085	MelanomaMouthOther
086	MelanomaNasalCavity
087	MelanomaNasopharynx
088	MelanomaOropharynx
089	MelanomaPalateHard
090	MelanomaPalateSoft
091	MelanomaPharynxOther
092	MelanomaSinusEthmoid
093	MelanomaSinusMaxillary
094	MelanomaSinusOther
095	MelanomaSkin
096	MelanomaTongueAnterior
097	MelanomaTongueBase
098	MerkelCellPenis

(Continued on next page)

(Continued from TNM 7/CS v0204+ (thru 2017))

Code	Description
099	MerkelCellScrotum
100	MerkelCellSkin
101	MerkelCellVulva
102	MiddleEar
103	MouthOther
104	MycosisFungoides
105	MyelomaPlasmaCellDisorder
106	NasalCavity
107	Nasopharynx
108	NETAmpulla
109	NETColon
110	NETRectum
111	NETSmallIntestine
112	NETStomach
113	Orbit
114	Oropharynx
115	Ovary
116	PalateHard
117	PalateSoft
118	PancreasBodyTail
119	PancreasHead
120	PancreasOther
121	ParotidGland
122	Penis
123	Peritoneum
124	PeritoneumFemaleGen
125	PharyngealTonsil
126	PharynxOther
127	Placenta
128	Pleura
129	Prostate
130	Rectum
131	RespiratoryOther
132	Retinoblastoma
133	Retroperitoneum

(Continued on next page)

(Continued from TNM 7/CS v0204+ (thru 2017)

Code	Description
134	SalivaryGlandOther
135	Scrotum
136	SinusEthmoid
137	SinusMaxillary
138	SinusOther
139	Skin
140	SkinEyelid
141	SmallIntestine
142	SoftTissue
143	Stomach
144	SubmandibularGland
145	Testis
146	Thyroid
147	TongueAnterior
148	TongueBase
149	Trachea
150	Urethra
151	UrinaryOther
152	Vagina
153	Vulva

TNM 7/CS V0204+ SCHEMA RECODE

NAACCR Item #: N/A
SAS Variable Name: TNM_7_CS_v0204_Schema_recode
Research: Yes
Research Limited-Field: No
Research Plus Limited-Field: No

Field Description: Allows the identification of categories consistent with TNM 7/CS v0204+ Schema to include cases 2018+. This variable will facilitate the analyses of the 2010+ SSDI recodes, which require consistent cohorts over time. Stomach and Esophagus GE Junction combined into a single grouping.

RACE RECODE (WHITE, BLACK, OTHER)

NAACCR Item #: N/A
SAS Variable Name: Race_recode_White_Black_Other
Research: Yes
Research Limited-Field: Yes
Research Plus Limited-Field: Yes

Field Description: Race recode is based on the race variables and the American Indian/Native American IHS link variable. This recode should be used to link to the populations for white, black and other. It is independent of Hispanic ethnicity. For more information, see http://seer.cancer.gov/seerstat/variables/seer/race_ethnicity.

Code	Description
1	White
2	Black
3	Other (American Indian/AK Native, Asian/Pacific Islander)
7	Other unspecified (1991+)
9	Unknown

RACE RECODE (W, B, AI, API)

NAACCR Item #: N/A
SAS Variable Name: Race_recode_W_B_AI_API
Research: Yes
Research Limited-Field: Yes
Research Plus Limited-Field: Yes

Field Description: Caution should be exercised when using this variable. For more information, see http://seer.cancer.gov/seerstat/variables/seer/race_ethnicity.

Code	Description
1	White
2	Black
3	American Indian/Alaska Native
4	Asian or Pacific Islander
7	Other unspecified (1991+)
9	Unknown

ORIGIN RECODE NHIA (HISPANIC, NON-HISP)

NAACCR Item #: N/A
SAS Variable Name: OriginrecodeNHIAHispanicNonHis
Research: Yes
Research Limited-Field: Yes
Research Plus Limited-Field: Yes

Field Description: Caution should be exercised when using this variable. For more information, see http://seer.cancer.gov/seerstat/variables/seer/race_ethnicity.

Code	Description
0	Non-Spanish-Hispanic-Latino
1	Spanish-Hispanic-Latino

RACE AND ORIGIN RECODE (NHW, NHB, NHAIAN, NHAPI, HISPANIC)

NAACCR Item #: N/A
SAS Variable Name: RaceandoriginrecodeNHWNHBNHAIAN
Research: Yes
Research Limited-Field: Yes
Research Plus Limited-Field: Yes

Field Description: Caution should be exercised when using this variable. For more information, see http://seer.cancer.gov/seerstat/variables/seer/race_ethnicity.

SEER HISTORIC STAGE A (1973-2015)

NAACCR Item #: N/A
SAS Variable Name: SEERhistoric_stage_A_1973_2015
Research: Yes
Research Limited-Field: No
Research Plus Limited-Field: No

Field Description: Derived from Collaborative Stage (CS) for 2004-2015 and Extent of Disease (EOD) from 1973-2003. It is a simplified version of stage: in situ, localized, regional, distant, & unknown. Over time several different EOD schemes have been used. Thus caution should be used when doing trend analysis.

For more information including sites and years for which it isn't calculated, see http://seer.cancer.gov/seerstat/variables/seer/lrd-stage.

Code	Description
0	In situ — A noninvasive neoplasm; a tumor which has not penetrated the basement membrane nor extended beyond the epithelial tissue. Some synonyms are intraepithelial (confined to epithelial tissue), noninvasive and noninfiltrating.
1	Localized — An invasive neoplasm confined entirely to the organ of origin. It may include intraluminal extension where specified. For example for colon, intraluminal extension limited to immediately contiguous segments of the large bowel is localized, if no lymph nodes are involved. Localized may exclude invasion of the serosa because of the poor survival of the patient once the serosa is invaded.
2	Regional — A neoplasm that has extended 1) beyond the limits of the organ of origin directly into surrounding organs or tissues; 2) into regional lymph nodes by way of the lymphatic system; or 3) by a combination of extension and regional lymph nodes.
4	Distant — A neoplasm that has spread to parts of the body remote from the primary tumor either by direct extension or by discontinuous metastasis (e.g., implantation or seeding) to distant organs, issues, or via the lymphatic system to distant lymph nodes.
8	Localized/Regional – Only used for Prostate cases.
9	Unstaged — Information is not sufficient to assign a stage.
14	Blank

AJCC STAGE 3rd EDITION (1988-2003)

NAACCR Item #: N/A
SAS Variable Name: AJCCstage3rd_edition_1988_2003
Research: Yes
Research Limited-Field: No
Research Plus Limited-Field: No

Field Description: Derived by algorithm from extent of disease (EOD). Not available for all years or for all sites.

For codes and descriptions, see:
http://seer.cancer.gov/seerstat/variables/seer/ajcc-stage/3rd.html

SEER MODIFIED AJCC STAGE 3rd ED (1988-2003)

NAACCR Item #: N/A
SAS Variable Name: SEERmodifiedAJCCstage3rd198820
Research: Yes
Research Limited-Field: No
Research Plus Limited-Field: No

Field Description: Derived by algorithm from extent of disease (EOD). Not available for all years or for all sites. The modified version stages cases that would be unstaged under strict AJCC staging rules. For example, it assumes NX is N0.

For codes and descriptions, see:
http://seer.cancer.gov/seerstat/variables/seer/ajcc-stage/3rd.html

FIRST MALIGNANT PRIMARY INDICATOR

NAACCR Item #: N/A
SAS Variable Name: Firstmalignantprimaryindicator
Research: Yes
Research Limited-Field: Yes
Research Plus Limited-Field: Yes

Field Description: Based on all the tumors in SEER. Tumors not reported to SEER are assumed malignant.

Code	Description
0	no
1	yes

STATE-COUNTY

NAACCR Item #: N/A
SAS Variable Name: N/A
Research: No
Research Limited-Field: No
Research Plus Limited-Field: Yes

Field Description: This item is a state-county combination where the first two characters represent the state FIPS code. The last three digits represent the FIPS county code.

Note: This data item is not available at the individual level.

COUNTY

NAACCR Item #: 90
SAS Variable Name: N/A
Research: No
Research Limited-Field: No
Research Plus Limited-Field: Yes

Field Description: County of residence at diagnosis. This must be used in conjunction with SEER registry or use State-county variable. For more information, see https://seer.cancer.gov/manuals/2004Revision%201/SPM_AppendixA.pdf.

Note: This data item is not available at the individual level.

COUNTY ATRRIBUTES

NAACCR Item #:
SAS Variable Names: Medianhouseholdincomeinflationa, Rural_Urban_Continuum_Code
Research: Yes
Research Limited-Field: Yes
Research Plus Limited-Field: Yes

Field Description: County attribute data from the U.S. Census are also linked to the SEER*Stat databases. Variables available are dependent on the type of database. For more information, see https://seer.cancer.gov/seerstat/variables/countyattribs/.
If using the linked attribute database: "County Attributes - Time Dependent (1990-2018) Income/Rurality, 1969-2019 Counties", which only includes 2 categorical attributes (income and rurality), these fields can be included in case listings. Research Plus and Research Plus Limited-Field databases also have additional linked county attribute databases available. If using these databases, the attributes are not available in case listing.

PRCDA 2017

NAACCR Item #: N/A
SAS Variable Name: PRCDA_2017
Research: Yes
Research Limited-Field: Yes
Research Plus Limited-Field: Yes

Field Description: This data item identifies whether or not the county of diagnosis is served by PRCDA. The primary use of this field is to be able to limit analyses of AI/AN race to areas served by PRCDA.
See https://seer.cancer.gov/seerstat/variables/seer/race_ethnicity
OR https://seer.cancer.gov/seerstat/variables/countyattribs/

PRCDA REGION

NAACCR Item #: N/A
SAS Variable Name: N/A
Research: No
Research Limited-Field: No
Research Plus Limited-Field: Yes

Field Description: This data item is a grouping of states that is primarily used when working with PRCDA 2017. See https://seer.cancer.gov/seerstat/variables/seer/race_ethnicity OR https://seer.cancer.gov/seerstat/variables/countyattribs/

Note: This data item is not available at the individual level.

Code	Description
1	Alaska
2	East
3	Northern Plains
5	Pacific Coast
6	Southwest

COD TO SITE RECODE

NAACCR Item #: N/A
SAS Variable Name: COD_to_site_recode
Research: Yes
Research Limited-Field: No
Research Plus Limited-Field: No

Field Description: This recode was introduced to account for several newly valid ICD-10 codes and includes both cancer and non-cancer causes of death.

See the following website for details:
http://seer.cancer.gov/codrecode/1969+_d09172004/index.html

COD TO SITE REC KM

NAACCR Item #: N/A
SAS Variable Name: COD_to_site_rec_KM
Research: Yes
Research Limited-Field: No
Research Plus Limited-Field: No

Field Description: This is a recode based on underlying cause of death to designate cause of death into groups similar to the incidence site recode with KS and mesothelioma. For more information, see http://seer.cancer.gov/codrecode/1969+_d04162012. Study cutoff date has been applied, i.e. coded as alive if death occurred after study cutoff.

VITAL STATUS RECODE (STUDY CUTOFF USED)

NAACCR Item #: N/A
SAS Variable Name: Vitalstatusrecodestudycutoffus
Research: Yes
Research Limited-Field: No
Research Plus Limited-Field: No

Field Description: Any patient that dies after the follow-up cut-off date is recoded to alive as of the cut-off date.

Code	Description
1	Alive
0	Dead

IHS LINK

NAACCR Item #: 192
SAS Variable Name: IHS_Link
Research: Yes
Research Limited-Field: Yes
Research Plus Limited-Field: Yes

Field Description: Incidence files are periodically linked with Indian Health Service (IHS) files to identify Native Americans. The race recode uses information from this field and race to determine if a person is Native American or not. See http://www.seer.cancer.gov/seerstat/variables/seer/race_ethnicity.

Code	Description
0	Record sent for linkage, no IHS match
1	Record sent for linkage, IHS match
2	Blank

SUMMARY STAGE 2000 (1998-2017)

NAACCR Item #: N/A
SAS Variable Name: Summary_stage_2000_1998_2017
Research: Yes
Research Limited-Field: No
Research Plus Limited-Field: No

Field Description: Summary Stage 2000 is derived from Collaborative Stage (CS) for 2004+ and Extent of Disease (EOD) from 1998-2003. It is a simplified version of stage: in situ, localized, regional, distant, & unknown. Used in the SEER CSR and more recent SEER publications. For more information including sites and years for which it isn't calculated, see http://seer.cancer.gov/seerstat/variables/seer/lrd-stage.

AYA SITE RECODE/WHO 2008

NAACCR Item #: N/A
SAS Variable Name: AYA_site_recode_WHO_2008
Research: Yes
Research Limited-Field: Yes
Research Plus Limited-Field: Yes

Field Description: A site/histology recode that is mainly used to analyze data on adolescent and young adults. The recode was applied to all cases no matter the age in order that age comparisons can be made with these groupings. For more information, see http://www.seer.cancer.gov/ayarecode/index.html.

AYA SITE RECODE 2020 REVISION

NAACCR Item #: N/A
SAS Variable Name: AYA_site_recode_2020_Revision
Research: Yes
Research Limited-Field: Yes
Research Plus Limited-Field: Yes

Field Description: A site/histology recode that is mainly used to analyze data on adolescent and young adults. The recode was applied to all cases no matter the age in order that age comparisons can be made with these groupings. For more information, see https://seer.cancer.gov/ayarecode/

LYMPHOID NEOPLASM RECODE 2021 REVISION

NAACCR Item #: N/A
SAS Variable Name: Lymphoidneoplasmrecode2021Revis
Research: Yes
Research Limited-Field: Yes
Research Plus Limited-Field: Yes

Field Description: A site/histology recode that is mainly used to analyze data on lymphoma subtypes. Based on ICD-O-3. Note that cases diagnosed before 2001 were not coded under ICD-O-3 and were converted to ICD-O-3 and may not have the specificity of cases after 2000 that were coded directly under ICD-O-3. For more information, see https://seer.cancer.gov/lymphomarecode/

LYMPHOMA SUBTYPE RECODE/WHO 2008 (thru 2017)

NAACCR Item #: N/A
SAS Variable Name: LymphomasubtyperecodeWHO2008thr
Research: Yes
Research Limited-Field: Yes
Research Plus Limited-Field: Yes

Field Description: A site/histology recode that is mainly used to analyze data on adolescent and young adults. The recode was applied to all cases no matter the age in order that age comparisons can be made with these groupings. For more information, see http://seer.cancer.gov/lymphomarecode/.

SEER BRAIN AND CNS RECODE

NAACCR Item #: N/A
SAS Variable Name: SEER_Brain_and_CNS_Recode
Research: Yes
Research Limited-Field: Yes
Research Plus Limited-Field: Yes

Field Description: For analyses of brain/CNS tumors according to major histological categories, based on the 2016 World Health Organization Classification of Tumors of the Central Nervous System (CNS). For more information, see https://seer.cancer.gov/seerstat/variables/seer/brain_cns-recode/

ICCC SITE RECODE 3RD EDITION/IARC 2017

NAACCR Item #: N/A
SAS Variable Name: ICCCsiterecode3rdeditionIARC20
Research: Yes
Research Limited-Field: Yes
Research Plus Limited-Field: Yes

Field Description: A site/histology recode that is mainly used to analyze data on children. The recode was applied to all cases no matter the age in order that age comparisons can be made with these groupings. Based on ICD-O-3. Note that cases diagnosed before 2001 were not coded under ICD-O-3 and were converted to ICD-O-3 and may not have the specificity of cases after 2000 that were coded directly under ICD-O-3. For more information on this International Classification of Childhood Cancer (ICCC) site recode, see https://seer.cancer.gov/iccc

SEER CAUSE-SPECIFIC DEATH CLASSIFICATION

NAACCR Item #: N/A
SAS Variable Name: SEERcausespecificdeathclassifi
Research: Yes
Research Limited-Field: No
Research Plus Limited-Field: No

Field Description: Created for use in cause-specific survival. This variable designates that the person died of their cancer for cause-specific survival. For more information, see http://seer.cancer.gov/causespecific.

Code	Description
0	Alive or dead of other cause
1	Dead
8	Dead (missing/unknown COD)
9	N/A

SEER OTHER CAUSE OF DEATH CLASSIFICATION

NAACCR Item #: N/A
SAS Variable Name: SEERothercauseofdeathclassific
Research: Yes
Research Limited-Field: No
Research Plus Limited-Field: No

Field Description: Created for use in left-truncated life table session. This variable designates that the person died of causes other than their cancer. For more information, see http://seer.cancer.gov/causespecific.

Code	Description
0	Alive or dead due to cancer
1	Dead
8	Dead (missing/unknown COD)
9	N/A

CS TUMOR SIZE/EXT EVAL (2004-2015)

NAACCR Item #: 2820
SAS Variable Name: CSTumorSize_Ext_Eval_2004_2015
Research: Yes
Research Limited-Field: No
Research Plus Limited-Field: No

Field Description: Available for 2004-2015, but not required for the entire timeframe. Will be blank in cases not collected. For more information, see http://seer.cancer.gov/seerstat/variables/seer/ajcc-stage.

CS REG NODE EVAL (2004-2015)

NAACCR Item #: 2840
SAS Variable Name: CS_Reg_Node_Eval_2004_2015
Research: Yes
Research Limited-Field: No
Research Plus Limited-Field: No

Field Description: Available for 2004-2015, but not required for the entire timeframe. Will be blank in cases not collected. For more information, see http://seer.cancer.gov/seerstat/variables/seer/ajcc-stage.

CS METS EVAL (2004-2015)

NAACCR Item #: 2860
SAS Variable Name: CS_Mets_Eval_2004_2015
Research: Yes
Research Limited-Field: No
Research Plus Limited-Field: No

Field Description: Available for 2004-2015,, but not required for the entire timeframe. Will be blank in cases not collected. For more information, see http://seer.cancer.gov/seerstat/variables/seer/ajcc-stage.

PRIMARY BY INTERNATIONAL RULES

NAACCR Item #: N/A
SAS Variable Name: Primary_by_international_rules
Research: Yes
Research Limited-Field: Yes
Research Plus Limited-Field: Yes

Field Description: Created using IARC multiple primary rules. Did not include benign tumors or non-bladder in situ tumors in algorithm. No tumor information was modified on any records.

Code	Description
0	No
1	Yes
9	Excluded from IARC multiple primary algorithm due to behavior

ER STATUS RECODE BREAST CANCER (1990+)

NAACCR Item #: N/A
SAS Variable Name: ERStatusRecodeBreastCancer1990
Research: Yes
Research Limited-Field: No
Research Plus Limited-Field: No

Field Description: Created with combined information from Tumor marker 1 (1990-2003) (NAACCR Item #1150), with information from CS site-specific factor 1 (2004-2017) (NAACCR Item #2880) and Estrogen Receptor Summary (2018+) (NAACCR Item #3827). Coded for cases coded as Breast in TNM 7/CS v0204+ Schema AND EOD Schema Id AND Site recode ICD-O-3/WHO 2008.

Code	Description
1	Positive
2	Negative
3	Borderline
4	Unknown
9	Recode Not Available

PR STATUS RECODE BREAST CANCER (1990+)

NAACCR Item #: N/A
SAS Variable Name: PRStatusRecodeBreastCancer1990
Research: Yes
Research Limited-Field: No
Research Plus Limited-Field: No

Field Description: Created with combined information from Tumor marker 2 (1990-2003) (NAACCR Item #1160), with information from CS site-specific factor 2 (2004-2017) (NAACCR Item #2890) and Progesterone Receptor Summary (2018+) (NAACCR Item #3915). Coded for cases coded as Breast in TNM 7/CS v0204+ Schema AND EOD Schema Id AND Site recode ICD-O-3/WHO 2008.

Code	Description
1	Positive
2	Negative
3	Borderline
4	Unknown
9	Recode Not Available

CS SCHEMA—AJCC 6TH EDITION

NAACCR Item #: N/A
SAS Variable Name: CS_Schema_AJCC_6th_Edition
Research: Yes
Research Limited-Field: No
Research Plus Limited-Field: No

Field Description: CS information is collected under the specifications of a particular schema based on site and histology. This recode should be used in any analysis of AJCC 6th ed stage and T, N, M. Based on CS version 1, it should not be used for SSFs collected or modified under CS v02.

Code	Description
01	LipUpper
02	LipLower
03	OthLip
04	BaseTongue
05	AntTongue
06	GumUpper
07	GumLower
08	OthGum
09	FOM
10	HardPalate
11	SoftPalate
12	OthMouth

(Continued on next page)

(Continued from CS SCHEMA—AJCC 6TH EDITION)

Code	Description
13	BuccalMucosa
14	ParotidGland
15	SubmandibularGland
16	OthSalivary
17	Oropharynx
18	AntEpiglottis
19	Nasopharynx
20	Hypopharynx
21	OthPharynx
22	Esophagus
23	Stomach
24	SmallIntestine
25	Colon
26	Rectum
27	Anus
28	Liver
29	Gallbladder
30	ExtraHepaticDucts
31	Ampulla
32	OthBiliary
33	PancreasHead
34	PancreasBodyTail
35	OthPancreas
36	OthDigestive
37	NasalCavity
38	MiddleEar
39	MaxillarySinus
40	EthmoidSinus
41	OthSinus
42	GlotticLarynx
43	SupraLarynx
44	SubLarynx
45	OthLarynx
46	Trachea
47	Lung
48	HeartMediastinum
49	Pleura
50	OthRespiratory
51	Bone

(Continued on next page)

(Continued from CS SCHEMA—AJCC 6TH EDITION)

Code	Description
52	Skin
53	SkinEyelid
54	Melanoma
55	MF
56	SoftTissue
57	Peritoneum
58	Breast
59	Vulva
60	Vagina
61	Cervix
62	Corpus
63	Ovary
64	FallopianTube
65	OthAdnexa
66	OthFemaleGen
67	Placenta
68	Penis
69	Prostate
70	Testis
71	OthMaleGen
72	Scrotum
73	Kidney
74	RenalPelvis
75	Bladder
76	Urethra
77	OthUrinary
78	Conjunctiva
79	MelanomaConjunctiva
80	OthEye
81	MelanomaIrisCiliary
82	MelanomaChoroid
83	MelanomaOthEye
84	LacrimalGland
85	Orbit
86	Retinoblastoma
87	Brain
88	OthCNS
89	Thyroid
90	OthEndocrine
91	KS
92	Lymphoma
93	HemeRetic
94	OthIllDef

LYMPH-VASCULAR INVASION (2004+ VARYING BY SCHEMA)

NAACCR Item #: 1182
SAS Variable Name: LymphvascularInvasion2004varyi
Research: Yes
Research Limited-Field: No
Research Plus Limited-Field: No

Field Description: LVI is required for cases originally coded under CSv2 or diagnosed 2010+ for the schemas for penis and testis only. On the research file LVI is shown only for testis because it is needed for AJCC 6th ed staging. For penis, LVI is needed for AJCC 7th ed staging only which isn't calculated until 2010.

Code	Description
0	Lymph-vascular Invasion Not Present (absent)/Not Identified
1	Lymph-vascular Invasion Present/Identified
8	Not Applicable
9	Unknown/Indeterminate
14	Blank

SURVIVAL MONTHS

NAACCR Item #: N/A
SAS Variable Name: Survival_months
Research: Yes
Research Limited-Field: No
Research Plus Limited-Field: No

Field Description: Created using complete dates, including days, therefore may differ from survival time calculated from year and month only. For more information, see http://seer.cancer.gov/survivaltime.

Code	Description
000-9998	000-9998 months
9999	Unknown

SURVIVAL MONTHS FLAG

NAACCR Item #: N/A
SAS Variable Name: Survival_months_flag
Research: Yes
Research Limited-Field: No
Research Plus Limited-Field: No

Field Description: Created using complete dates, including days, therefore may differ from survival time calculated from year and month only. For more information, see http://seer.cancer.gov/survivaltime.

Code	Description
0	Complete dates are available and there are 0 days of survival
1	Complete dates are available and there are more than 0 days of survival
2	Incomplete dates are available and there could be zero days of follow-up
3	Incomplete dates are available and there cannot be zero days of follow-up
8	Not calculated because a Death Certificate Only or Autopsy Only case
9	Unknown

DERIVED AJCC T, 7TH ED (2010-2015)

NAACCR Item #: 3400
SAS Variable Name: DerivedAJCC_T_7th_ed_2010_2015
Research: Yes
Research Limited-Field: No
Research Plus Limited-Field: No

Field Description: This is the AJCC "T" component that is derived from CS coded fields, using the CS algorithm, effective with 2010-2015 diagnosis years. See the CS site-specific schema for details: (http://seer.cancer.gov/seerstat/variables/seer/ajcc-stage).

Code	Description
999	TX
000	T0
010	Ta
050	Tis
060	Tispu (Urethra only)
070	Tispd (Urethra only)
100	T1
110	T1mic
199	T1 NOS
120	T1a
130	T1a1
140	T1a2
150	T1b
160	T1b1
170	T1b2
180	T1c
181	T1d
200	T2
299	T2 NOS
210	T2a
211	T2a1
212	T2a2
213	T2a NOS
220	T2b
230	T2c
240	T2d
300	T3
399	T3 NOS
310	T3a
320	T3b
330	T3c
400	T4
499	T4 NOS

(Continued on next page)

(Continued from DERIVED AJCC T, 7TH ED (2010-2015))

Code	Description
410	T4a
420	T4b
430	T4c
440	T4d
450	T4e
800	T1a NOS
810	T1b NOS
888	Not applicable
1022	Blank

DERIVED AJCC N, 7TH ED (2010-2015)

NAACCR Item #: 3410
SAS Variable Name: DerivedAJCC_N_7th_ed_2010_2015
Research: Yes
Research Limited-Field: No
Research Plus Limited-Field: No

Field Description: This is the AJCC "N" component that is derived from CS coded fields, using the CS algorithm, effective with 2010-2015 diagnosis years. See the CS site-specific schema for details: (http://seer.cancer.gov/seerstat/variables/seer/ajcc-stage).

**Code**	**Description**
999	NX
000	N0
010	N0(i-)
020	N0(i+)
030	N0(mol-)
040	N0(mol+)
100	N1
199	N1 NOS
110	N1a
120	N1b
130	N1c
180	N1mi
200	N2
299	N2 NOS
210	N2a
220	N2b
230	N2c
300	N3

(Continued on next page)

(Continued from DERIVED AJCC N, 7TH ED (2010-2015))

Code	Description
399	N3 NOS
310	N3a
320	N3b
330	N3c
400	N4
888	Not applicable
1022	Blank

DERIVED AJCC M, 7TH ED (2010-2015)

NAACCR Item #: 3420
SAS Variable Name: DerivedAJCC_M_7th_ed_2010_2015
Research: Yes
Research Limited-Field: No
Research Plus Limited-Field: No

Field Description: This is the AJCC "M" component that is derived from CS coded fields, using the CS algorithm, effective with 2010-2015 diagnosis years. See the CS site-specific schema for details: (http://seer.cancer.gov/seerstat/variables/seer/ajcc-stage).

Code	Description
999	MX
000	M0
010	M0(i+)
100	M1
110	M1a
120	M1b
130	M1c
140	M1d
150	M1e
199	M1 NOS
888	Not applicable
1022	Blank

DERIVED AJCC STAGE GROUP, 7TH ED (2010-2015)

NAACCR Item #: 3430
SAS Variable Name: DerivedAJCCStageGroup7thed2010
Research: Yes
Research Limited-Field: No
Research Plus Limited-Field: No

Field Description: This is the AJCC "Stage Group" component that is derived from CS coded fields, using the CS algorithm, effective with 2010-2015 diagnosis years. See the CS site-specific schema for details: (http://seer.cancer.gov/seerstat/variables/seer/ajcc-stage).

Code	Description
000	Stage 0
010	Stage 0a
020	Stage 0is
100	Stage I
110	Stage I NOS
120	Stage IA
130	Stage IA1
140	Stage IA2
121	Stage IA NOS
150	Stage IB
160	Stage IB1
170	Stage IB2
151	Stage IB NOS
180	Stage IC
190	Stage IS
230	Stage ISA (lymphoma only)
240	Stage ISB (lymphoma only)
200	Stage IEA (lymphoma only)
210	Stage IEB (lymphoma only)
220	Stage IE (lymphoma only)
300	Stage II
310	Stage II NOS
320	Stage IIA
321	Stage IIA NOS
322	Stage IIA1
323	Stage IIA NOS
330	Stage IIB
340	Stage IIC
350	Stage IIEA (lymphoma only)
360	Stage IIEB (lymphoma only)

(Continued on next page)

(Continued from DERIVED AJCC STAGE GROUP, 7TH ED (2010-2015)

Code	Description
370	Stage IIE (lymphoma only)
380	Stage IISA (lymphoma only)
390	Stage IISB (lymphoma only)
400	Stage IIS (lymphoma only)
410	Stage IIESA (lymphoma only)
420	Stage IIESB (lymphoma only)
430	Stage IIES (lymphoma only)
500	Stage III
510	Stage III NOS
520	Stage IIIA
530	Stage IIIB
540	Stage IIIC
541	Stage IIIC1
542	Stage IIIC2
550	Stage IIIEA (lymphoma only)
560	Stage IIIEB (lymphoma only)
570	Stage IIIE (lymphoma only)
580	Stage IIISA (lymphoma only)
590	Stage IIISB (lymphoma only)
600	Stage IIIS (lymphoma only)
610	Stage IIIESA (lymphoma only)
620	Stage IIIESB (lymphoma only)
630	Stage IIIES (lymphoma only)
700	Stage IV
710	Stage IV NOS
720	Stage IVA
730	Stage IVB
740	Stage IVC
888	Not applicable
900	Stage Occult
999	Stage Unknown
1022	Blank

BREAST—ADJUSTED AJCC 6TH T (1988-2015)

NAACCR Item #: N/A
SAS Variable Name: BreastAdjustedAJCC6thT19882015
Research: Yes
Research Limited-Field: No
Research Plus Limited-Field: No

Field Description: Created from merged EOD 3rd Edition and Collaborative Stage disease information. Currently only available for Breast schema. For more information see http://seer.cancer.gov/seerstat/variables/seer/ajcc-stage/6th.

Code	Description
00	T0
01	Ta
05	Tis
06	Tispu
07	Tispd
10	T1
11	T1mic
12	T1a
13	T1a1
14	T1a2
15	T1b
16	T1b1
17	T1b2
18	T1c
19	T1NOS
20	T2
21	T2a
22	T2b
23	T2c
29	T2NOS
30	T3
31	T3a
32	T3b
33	T3c
39	T3NOS
40	T4
41	T4a
42	T4b
43	T4c
44	T4d
49	T4NOS
60	Any T, Mets
80	T1aNOS

(Continued on next page)

(Continued from BREAST—ADJUSTED AJCC 6TH T (1988-2015)

Code	Description
81	T1bNOS
88	N/A
99	TX Adjusted
126	Blank

BREAST—ADJUSTED AJCC 6TH N (1988-2015)

NAACCR Item #: N/A
SAS Variable Name: BreastAdjustedAJCC6thN19882015
Research: Yes
Research Limited-Field: No
Research Plus Limited-Field: No

Field Description: Created from merged EOD 3rd Edition and Collaborative Stage disease information. Currently only available for Breast schema. For more information see http://seer.cancer.gov/seerstat/variables/seer/ajcc-stage/6th.

Code	Description
00	N0
01	N0(i-)
02	N0(i+)
03	N0(mo1-)
04	N0(mo1+)
09	N0NOS
10	N1
11	N1a
12	N1b
13	N1c
18	N1mi
19	N1NOS
20	N2
21	N2a
22	N2b
23	N2c
29	N2NOS
30	N3
31	N3a

(Continued on next page)

(Continued from BREAST—ADJUSTED AJCC 6TH N (1988-2015))

Code	Description
32	N3b
33	N3c
39	N3NOS
60	Any N, Mets
70	NXr
80	NXa
88	N/A
99	NX Adjusted
126	Blank

BREAST—ADJUSTED AJCC 6TH M (1988-2015)

NAACCR Item #: N/A
SAS Variable Name: BreastAdjustedAJCC6thM19882015
Research: Yes
Research Limited-Field: No
Research Plus Limited-Field: No

Field Description: Created from merged EOD 3rd Edition and Collaborative Stage disease information. Currently only available for Breast schema. For more information see http://seer.cancer.gov/seerstat/variables/seer/ajcc-stage/6th.

Code	Description
00	M0
10	M1
11	M1a
12	M1b
13	M1c
19	M1NOS
88	N/A
99	MX
126	Blank

BREAST—ADJUSTED AJCC 6TH STAGE (1988-2015)

NAACCR Item #: N/A
SAS Variable Name: BreastAdjustedAJCC6thStage1988
Research: Yes
Research Limited-Field: No
Research Plus Limited-Field: No

Field Description: Created from merged EOD 3rd Edition and Collaborative Stage disease information. Currently only available for Breast schema. For more information see http://seer.cancer.gov/seerstat/variables/seer/ajcc-stage/6th.

Code	Description
00	0
01	0a
02	0is
10	I
11	INOS
12	IA
13	IA1
14	IA2
15	IB
16	IB1
17	IB2
18	IC
19	IS
20	IEA
21	IEB
22	IE
23	ISA
24	ISB
30	II
31	IINOS
32	IIA
33	IIB
34	IIC
35	IIEA
36	IIEB
37	IIE
38	IISA
39	IISB
40	IIS
41	IIESA

(Continued on next page)

(Continued from BREAST—ADJUSTED AJCC 6TH STAGE (1988-2015))

Code	Description
43	IIES
50	III
51	IIINOS
52	IIIA
53	IIIB
54	IIIC
55	IIIEA
56	IIIEB
57	IIIE
58	IIISA
59	IIISB
60	IIIS
61	IIIESA
62	IIIESB
63	IIIES
70	IV
71	IVNOS
72	IVA
73	IVB
74	IVC
88	N/A
90	OCCULT
99	UNK Stage
126	Blank

DERIVED HER2 RECODE (2010+)

NAACCR Item #: N/A
SAS Variable Name: Derived_HER2_Recode_2010
Research: Yes
Research Limited-Field: No
Research Plus Limited-Field: No

Field Description: Created with combined information from several CS site-specific factors (2010-2017) and HER2 Overall Summary (2018+) (NAACCR Item #=3855). For more information, see https://seer.cancer.gov/seerstat/databases/ssf/her2-derived.html. Coded for cases coded as Breast in TNM 7/CS v0204+ Schema AND EOD Schema Id.

Code	Description
1	Positive
2	Negative
3	Borderline
4	Unknown
9	Recode Not Available

BREAST SUBTYPE (2010+)

NAACCR Item #: N/A
SAS Variable Name: Breast_Subtype_2010
Research: Yes
Research Limited-Field: Yes
Research Plus Limited-Field: Yes

Field Description: Created with combined information from ER Status Recode Breast Cancer (1990+), PR Status Recode Breast Cancer (1990+), and Derived HER2 Recode (2010+). For more information, see https://seer.cancer.gov/seerstat/databases/ssf/breast-subtype.html. Coded for cases coded as Breast in TNM 7/CS v0204+ Schema AND EOD Schema Id.

Code	Description
1	Her2+/HR+
2	Her2+/HR-
3	Her2-/HR+
4	Triple Negative
5	Unknown
9	Recode Not Available

LYMPHOMAS: ANN ARBOR STAGING (1983-2015)

NAACCR Item #: N/A
SAS Variable Name: LymphomaAnnArborStage1983_2015
Research: Yes
Research Limited-Field: No
Research Plus Limited-Field: No

Field Description: For information on how this field is created, visit http://seer.cancer.gov/seerstat/variables/seer/ajcc-stage/ann-arbor/

SEER COMBINED METS AT DX-BONE (2010+)

NAACCR Item #: N/A
SAS Variable Name: SEERCombinedMetsatDX_bone_2010
Research: Yes
Research Limited-Field: No
Research Plus Limited-Field: No

Field Description: Created from NAACR Item #2851 (2010-2015) and #1112 (2016+).

Code	Description
0	None; no bone metastases
1	Yes
8	Not applicable
9	Unknown; not documented in patient record
14	Blank

SEER COMBINED METS AT DX-BRAIN (2010+)

NAACCR Item #: N/A
SAS Variable Name: SEERCombinedMetsatDXbrain_2010
Research: Yes
Research Limited-Field: No
Research Plus Limited-Field: No

Field Description: Created from NAACR Item #2852 (2010-2015) and #1113 (2016+).

Code	Description
0	None; no brain metastases
1	Yes
8	Not applicable
9	Unknown; not documented in patient record
14	Blank

SEER COMBINED METS AT DX-LIVER (2010+)

NAACCR Item #: N/A
SAS Variable Name: SEERCombinedMetsatDXliver_2010
Research: Yes
Research Limited-Field: No
Research Plus Limited-Field: No

Field Description: Created from NAACR Item #2853 (2010-2015) and #1115 (2016+).

Code	Description
0	None; no liver metastases
1	Yes
8	Not applicable
9	Unknown; not documented in patient record
14	Blank

SEER COMBINED METS AT DX-LUNG (2010+)

NAACCR Item #: N/A
SAS Variable Name: SEERCombinedMetsatDX_lung_2010
Research: Yes
Research Limited-Field: No
Research Plus Limited-Field: No

Field Description: Created from NAACR Item #2854 (2010-2015) and #1116 (2016+).

Code	Description
0	None; no lung metastases
1	Yes
8	Not applicable
9	Unknown; not documented in patient record
14	Blank

T VALUE - BASED ON AJCC 3rd (1988-2003)

NAACCR Item #: N/A
SAS Variable Name: TvaluebasedonAJCC3rd_1988_2003
Research: Yes
Research Limited-Field: No
Research Plus Limited-Field: No

Field Description: Derived by algorithm from extent of disease (EOD). Not available for all years or for all sites.

For codes and descriptions, see:
http://seer.cancer.gov/seerstat/variables/seer/ajcc-stage/3rd.html

N VALUE - BASED ON AJCC 3rd (1988-2003)

NAACCR Item #: N/A
SAS Variable Name: NvaluebasedonAJCC3rd_1988_2003
Research: Yes
Research Limited-Field: No
Research Plus Limited-Field: No

Field Description: Derived by algorithm from extent of disease (EOD). Not available for all years or for all sites.

For codes and descriptions, see:
http://seer.cancer.gov/seerstat/variables/seer/ajcc-stage/3rd.html

M VALUE - BASED ON AJCC 3rd (1988-2003)

NAACCR Item #: N/A
SAS Variable Name: MvaluebasedonAJCC3rd_1988_2003
Research: Yes
Research Limited-Field: No
Research Plus Limited-Field: No

Field Description: Derived by algorithm from extent of disease (EOD). Not available for all years or for all sites.

For codes and descriptions, see:
http://seer.cancer.gov/seerstat/variables/seer/ajcc-stage/3rd.html

TOTAL NUMBER OF IN SITU/MALIGNANT TUMORS FOR PATIENT

NAACCR Item #: N/A
SAS Variable Name: Totalnumberofinsitumalignanttu
Research: Yes
Research Limited-Field: No
Research Plus Limited-Field: No

Field Description: Count of a patient's total reported in situ/malignant cancers, based on maximum sequence number of any in situ/malignant tumors in SEER through the last released year of diagnosis. This value is the same across all tumors for a person.

Valid values: 00-98; 99 (unknown)

TOTAL NUMBER OF BENIGN/BORDERLINE TUMORS FOR PATIENT

NAACCR Item #: N/A
SAS Variable Name: Totalnumberofbenignborderlinet
Research: Yes
Research Limited-Field: No
Research Plus Limited-Field: No

Field Description: Count of a patient's total reported benign/borderline cancers, based on maximum sequence number of any benign/borderline tumors in SEER through the last released year of diagnosis. This value is the same across all tumors for a person.

Valid values: 00-98; 99 (unknown)

RADIATION TO BRAIN OR CNS RECODE (1988-1997)

NAACCR Item #: N/A
SAS Variable Name: RadiationtoBrainorCNSRecode1988
Research: No
Research Limited-Field: No
Research Plus Limited-Field: No

Field Description: This variable was only collected for years 1988-1997 for lung and leukemia cases only. It codes for radiation given to the brain or central nervous system at all facilities as part of the first course of therapy, and is a recode of NAACCR Item #1370. This field is blank for cases diagnosed prior to 1988 and after 1997.

Code	Description
0	None/Unknown
1	Radiation
7	Refused
8	Recommended, unknown if administered

TUMOR SIZE SUMMARY (2016+)

NAACCR Item #: 756
SAS Variable Name: Tumor_Size_Summary_2016
Research: Yes
Research Limited-Field: No
Research Plus Limited-Field: No

Field Description: The most accurate measurement of a solid primary tumor, usually measured on the surgical resection specimen.

Code	Description
000	No mass/tumor found
001	1 mm or described as less than 1 mm (0.1 cm or less than 0.1 cm)
002-988	Exact size in millimeters (2mm-988mm) (0.2 cm to 98.8 cm)
989	989 millimeters or larger (98.9 cm or larger)
990	Microscopic focus or foci only and no size of focus is given
998	Alternate descriptions of tumor size for specific sites
999	Unknown; size not stated; Not documented in patient record; Size of tumor cannot be assessed; No excisional biopsy or tumor resection done; The only measurement(s) describes pieces or chips; Not applicable
1022	Blank

DERIVED SEER CMB STG GRP (2016-2017)

NAACCR Item #: 3614
SAS Variable Name: DerivedSEERCmbStg_Grp_2016_2017
Research: Yes
Research Limited-Field: No
Research Plus Limited-Field: No

Field Description: The results of the derived algorithmic calculation of Derived SEER Combined Stage Group.

DERIVED SEER COMBINED T (2016-2017)

NAACCR Item #: 3616
SAS Variable Name: DerivedSEERCombined_T_2016_2017
Research: Yes
Research Limited-Field: No
Research Plus Limited-Field: No

Field Description: The results of the derived algorithmic calculation of Derived SEER Combined T.

DERIVED SEER COMBINED N (2016-2017)

NAACCR Item #: 3618
SAS Variable Name: DerivedSEERCombined_N_2016_2017
Research: Yes
Research Limited-Field: No
Research Plus Limited-Field: No

Field Description: The results of the derived algorithmic calculation of Derived SEER Combined N.

DERIVED SEER COMBINED M (2016-2017)

NAACCR Item #: 3620
SAS Variable Name: DerivedSEERCombined_M_2016_2017
Research: Yes
Research Limited-Field: No
Research Plus Limited-Field: No

Field Description: The results of the derived algorithmic calculation of Derived SEER Combined M.

DERIVED SEER COMBINED T SRC (2016-2017)

NAACCR Item #: 3622
SAS Variable Name: DerivedSEERCombinedTSrc20162017
Research: Yes
Research Limited-Field: No
Research Plus Limited-Field: No

Field Description: The source information selected for the derived algorithmic calculation of Derived SEER Combined T #3616.

Code	Description
1	Clinical
2	Pathologic
3	Clinical and pathologic information used
9	Unknown
14	Blank

DERIVED SEER COMBINED N SRC (2016-2017)

NAACCR Item #: 3624
SAS Variable Name: DerivedSEERCombinedNSrc20162017
Research: Yes
Research Limited-Field: No
Research Plus Limited-Field: No

Field Description: The source information selected for the derived algorithmic calculation of Derived SEER Combined N #3618.

Code	Description
1	Clinical
2	Pathologic
3	Clinical and pathologic information used
9	Unknown
14	Blank

DERIVED SEER COMBINED M SRC (2016-2017)

NAACCR Item #: 3626
SAS Variable Name: DerivedSEERCombinedMSrc20162017
Research: Yes
Research Limited-Field: No
Research Plus Limited-Field: No

Field Description: The source information selected for the derived algorithmic calculation of Derived SEER Combined M #3620.

Code	Description
1	Clinical
2	Pathologic
3	Clinical and pathologic information used
9	Unknown
14	Blank

TNM EDITION NUMBER (2016-2017)

NAACCR Item #: 1060
SAS Variable Name: TNM_Edition_Number_2016_2017
Research: Yes
Research Limited-Field: No
Research Plus Limited-Field: No

Field Description: The edition of the AJCC manual used to stage the case. This applies to the manually coded AJCC fields. It does not apply to the Derived AJCC T, N, M and AJCC Stage Group fields [2940, 2960, 2980, and 3000].

SEER used UICC 7th edition as well as AJCC 7th edition rules. UICC 7th edition and AJCC 7th edition TNM categories and stage groups are very similar.

Code	Description
07	Seventh Edition (published 2009), recommended for use with cases diagnosed 2010-2017
U7	Seventh Edition with UICC modifications
88	Not applicable (cases that do not have an AJCC staging scheme)
14	Blank

METS AT DX—DISTANT LN (2016+)

NAACCR Item #: 1114
SAS Variable Name: Mets_at_DX_Distant_LN_2016
Research: Yes
Research Limited-Field: No
Research Plus Limited-Field: No

Field Description: Identifies whether distant lymph node(s) are an involved metastatic site.

Code	Description
0	None; no distant lymph node metastases
1	Yes; distant lymph node metastases
8	Not applicable
9	Unknown whether distant lymph node(s) are involved metastatic site. Not documented in patient record.
14	Blank

METS AT DX—OTHER (2016+)

NAACCR Item #: 1117
SAS Variable Name: Mets_at_DX_Other_2016
Research: Yes
Research Limited-Field: No
Research Plus Limited-Field: No

Field Description: Identifies any type of distant involvement not captured in the Mets at Dx-Bone [1112], Mets at Dx-Brain [1113], Mets at Dx-Liver [1115], Mets at Dx-Lung [1116], and Mets at Dx-Distant LN [1114] fields. It includes involvement of other specific sites and more generalized metastases such as carcinomatosis. Some examples include but are not limited to the adrenal gland, bone marrow, pleura, malignant pleural effusion, peritoneum, and skin.

Code	Description
0	None; no other metastases
1	Yes; distant metastases in known site(s) other than bone, brain, liver, lung or distant lymph nodes (Note: includes bone marrow involvement for lymphomas)
2	Generalized metastases such as carcinomatosis
8	Not applicable
9	Unknown whether any other metastatic site or generalized metastases. Not documented in patient record.
14	Blank

AJCC ID (2018+)

NAACCR Item #: 995
SAS Variable Name: AJCC_ID_2018
Research: Yes
Research Limited-Field: Yes
Research Plus Limited-Field: Yes

Field Description: Based on the chapters of the AJCC manual and will be derived primarily from the site/histology fields and other data items as required. IDs are assigned to cases for which AJCC staging is applicable. When staging is not applicable, code ‘XX’ is used.

EOD SCHEMA ID RECODE (2010+)

NAACCR Item #: N/A
SAS Variable Name: EOD_Schema_ID_Recode_2010
Research: Yes
Research Limited-Field: Yes
Research Plus Limited-Field: Yes

Field Description: Allows the identification of categories consistent with Schema ID to include cases 2010+. This variable will facilitate the analyses of the 2010+ SSDI recodes, which require consistent cohorts over time.

DERIVED EOD 2018 T (2018+)

NAACCR Item #: 785
SAS Variable Name: Derived_EOD_2018_T_2018
Research: Yes
Research Limited-Field: No
Research Plus Limited-Field: No

Field Description: See the most current version of EOD (https://staging.seer.cancer.gov/) for rules and site-specific codes and coding structures.

DERIVED EOD 2018 N (2018+)

NAACCR Item #: 815
SAS Variable Name: Derived_EOD_2018_N_2018
Research: Yes
Research Limited-Field: No
Research Plus Limited-Field: No

Field Description: See the most current version of EOD (https://staging.seer.cancer.gov/) for rules and site-specific codes and coding structures.

DERIVED EOD 2018 M (2018+)

NAACCR Item #: 795
SAS Variable Name: Derived_EOD_2018_M_2018
Research: Yes
Research Limited-Field: No
Research Plus Limited-Field: No

Field Description: See the most current version of EOD (https://staging.seer.cancer.gov/) for rules and site-specific codes and coding structures.

DERIVED EOD 2018 STAGE GROUP (2018+)

NAACCR Item #: 818
SAS Variable Name: DerivedEOD2018_Stage_Group_2018
Research: Yes
Research Limited-Field: No
Research Plus Limited-Field: No

Field Description: See the most current version of EOD (https://staging.seer.cancer.gov/) for rules and site-specific codes and coding structures.

EOD PRIMARY TUMOR (2018+)

NAACCR Item #: 772
SAS Variable Name: EOD_Primary_Tumor_2018
Research: Yes
Research Limited-Field: No
Research Plus Limited-Field: No

Field Description: See the most current version of EOD (https://staging.seer.cancer.gov/) for rules and site-specific codes and coding structures.

EOD REGIONAL NODES (2018+)

NAACCR Item #: 774
SAS Variable Name: EOD_Regional_Nodes_2018
Research: Yes
Research Limited-Field: No
Research Plus Limited-Field: No

Field Description: See the most current version of EOD (https://staging.seer.cancer.gov/) for rules and site-specific codes and coding structures.

EOD METS (2018+)

NAACCR Item #: 776
SAS Variable Name: EOD_Mets_2018
Research: Yes
Research Limited-Field: No
Research Plus Limited-Field: No

Field Description: See the most current version of EOD (https://staging.seer.cancer.gov/) for rules and site-specific codes and coding structures.

MONTHS FROM DIAGNOSIS TO TREATMENT

NAACCR Item #: N/A
SAS Variable Name: Monthsfromdiagnosisto_treatment
Research: No
Research Limited-Field: No
Research Plus Limited-Field: No

Field Description: Value = ((Year initial treatment started * 12) + Month initial treatment started) - ((Year of DX * 12) + Month of DX);

Blank if any date component unknown or calculated value > 24
