# Supplementary material for: Chemotherapy and Heart-Specific Mortality in Elderly Men with Prostate Cancer: A Propensity Score Matching Analysis
Source: PLoS One. 2025 Apr 11;20(4):e0318429. doi: 10.1371/journal.pone.0318429 (PMC11990641; doi:10.1371/journal.pone.0318429)
Supplement: Attachment — Submitted filename: Response_to_Reviewers_auresp_3.docx [file pone.0318429.s007.docx]

**Reviewer #1**

The authors have repeatedly indicated that there are inherent limitation of the database they used for the study, however, this does effectively address the issue the cohort selection and characterization appeared in the result section. When comparing chemo vs non-chemo patients, the author's failed to address the the chemo group also received other therapies and if they were comparable to the non-chemo patients. Since the authors can extract the chemo treatment record from the database, it is naturally to assume that the authors can evaluate other therapy records as well.

**Response:**

**Dear reviewer, thank you for your question. In the SEER database, the treatment of prostate cancer patients mainly includes chemotherapy and radiotherapy. Therefore, these are also all the treatment methods available to us for elderly PC. We have uploaded the original data form used for the analysis of the paper after cleaning, hoping to obtain your understanding. At the same time, when comparing the patients in the chemotherapy and non-chemotherapy groups, we used propensity matching. As shown in Table I, after propensity matching, the total number of patients in the chemotherapy group and the non-chemotherapy group was 1361, the number of patients in the non-chemotherapy group was 158 (11.6%), while the number of patients in the chemotherapy group received radiotherapy was 141 (10.4%). There was a high degree of agreement between the two groups with respect to other baseline measures. We can also observe this result intuitively from Figure 3. Thus, the chemotherapy and non-chemotherapy groups were consistent in terms of the treatment received. We have highlighted the chart in yellow to facilitate your review. I hope our explanation can meet your requirements. Thank you!**

The LOT of the chemotherapy in the chemo group and LOT of non-chemo in non-chemo group should also be considered.

**Response:**

**Dear reviewer, thank you for your question. As you said, what you are focusing on is exactly what we are worried about. In order to avoid the influence of lead time bias on survival time. All The Times in our analyses refer to the time of initial center diagnosis and initiation of treatment, not to the time of screening detection. In addition, our data are all from SEER database, and the inclusion criteria of data are consistent, so the bias caused by LOT can be basically avoided. But according to your suggestion, We have expanded the discussion section to include a more detailed consideration of Lead Time Bias and its potential impact on our study findings. We have also discussed the methods we employed to mitigate this bias and the implications of our approach for the interpretation of the results.**

**We also added the corresponding content in the limitation part of the discussion, and marked it with a yellow highlight background. I hope our modification can meet your requirements. Thank you!**

**References:**

**[1]Bartkowiak D, Thamm R, Siegmann A, Böhmer D, Budach V, Wiegel T. Lead-time bias does not falsify the efficacy of early salvage radiotherapy for recurrent prostate cancer. Radiother Oncol. 2021 Jan;154:255-259. doi: 10.1016/j.radonc.2020.09.009. Epub 2020 Sep 11. PMID: 32920006.**

**[2]Benbassat J. Estimates of the lead time in screening for bladder cancer. Urol Oncol. 2024 Apr;42(4):110-114. doi: 10.1016/j.urolonc.2023.11.013. Epub 2024 Jan 16. PMID: 38514215.**

**[3]Daher D, Seif El Dahan K, Rich NE, Tayob N, Merrill V, Huang DQ, Yang JD, Kulkarni AV, Kanwal F, Marrero J, Parikh N, Singal AG. Hepatocellular Carcinoma Screening in a Contemporary Cohort of At-Risk Patients. JAMA Netw Open. 2024 Apr 1;7(4):e248755. doi: 10.1001/jamanetworkopen.2024.8755. PMID: 38683607; PMCID: PMC11059036.**

**[4]Ma Z, Wang Z, Li Y, Zhang Y, Chen H. Detection and treatment of lung adenocarcinoma at pre-/minimally invasive stage: is it lead-time bias? J Cancer Res Clin Oncol. 2022 Oct;148(10):2717-2722. doi: 10.1007/s00432-022-04031-z. Epub 2022 May 7. PMID: 35524781.**

**Reviewer #3**

I feel that the impact of confounding factors is not adequately addressed.
I understand that this is a difficult issue, as many diseases may be taken into account, but it is an important point as there is a possibility that the interpretation of the study results may become arbitrary.

**Response:**

**Dear Reviewer,**

**Thank you for your insightful comments and for highlighting the importance of addressing confounding factors in our study. We understand your concern about the potential impact of these factors on the interpretation of our results. We have taken your feedback seriously and have made additional efforts to further address the impact of confounding factors in our analysis. Below is a detailed response on how we have revised our manuscript to better account for these issues.**

**Enhanced Propensity Score Matching (PSM):**

**We have refined our propensity score matching methodology to more accurately balance the confounding factors between the chemotherapy and non-chemotherapy groups. This includes not only demographic and clinical characteristics but also the inclusion of additional covariates that may influence treatment outcomes, such as comorbidities and lifestyle factors, to the extent that these data are available in the SEER database.**

**Sensitivity Analyses:**

**To further validate our findings, we have conducted additional sensitivity analyses. These include analyses with different matching ratios (1:2 and 1:4) and the use of inverse probability of treatment weighting (IPTW) to assess the robustness of our results under various assumptions about the treatment assignment mechanism.**

**Stratified Analysis:**

**We have performed stratified analyses based on key confounders to assess the consistency of our findings across different subgroups. This approach allows us to explore potential effect modifiers and to ensure that our results are not driven by a particular subgroup of patients.**

**Multivariate Competing Risk Regression (CRR) Models:**

**We have expanded our multivariate CRR models to include more covariates that could potentially confound the relationship between chemotherapy and heart-specific mortality (HSM). These models now adjust for a broader range of clinical and pathological factors, as well as treatment-related variables, to provide a more comprehensive assessment of the independent effects of chemotherapy on HSM.**

**Discussion of Unmeasured Confounders:**

**In the discussion section, we have added a more detailed consideration of potential unmeasured confounders and their potential impact on our findings. We acknowledge the limitations inherent in observational studies and the potential for residual confounding despite our best efforts to control for known confounders.**

**We add again in the discussion section of the article the efforts we have made to ensure that the results are actionable. We have annotated in red font in the hope that our modifications will meet your requirements: "However, our study enhanced the PSM method to more accurately balance confounding factors between chemotherapy and non-chemotherapy groups. We also performed additional sensitivity analyses including different matching ratios (1:2 and 1:4) and IPTW using treatment weights to assess the robustness of our results under different assumptions. In addition, we performed stratified analyses based on key confounders to ensure that our findings were consistent across subgroups. Our CRR model now adjusts for a wider range of clinical and pathological factors, as well as treatment-related variables, to provide a more comprehensive assessment of the independent effects of chemotherapy on HSM. Therefore, our conclusion is highly feasible."**

**Finally, we ensured that all methods, analyses, and results related to the handling of confounding factors were transparently and thoroughly reported, in adherence with the STROBE (Strengthening the Reporting of Observational Studies in Epidemiology) guidelines, allowing for a clear understanding of our methodology and its potential limitations.**

**We believe that these revisions have significantly strengthened our manuscript by addressing the impact of confounding factors more comprehensively. We appreciate your guidance and the opportunity to improve the quality and robustness of our research.**
