# Supplementary material for: Chemotherapy and Heart-Specific Mortality in Elderly Men with Prostate Cancer: A Propensity Score Matching Analysis
Source: PLoS One. 2025 Apr 11;20(4):e0318429. doi: 10.1371/journal.pone.0318429 (PMC11990641; doi:10.1371/journal.pone.0318429)
Supplement: Attachment — Submitted filename: Response to Reviewers.docx [file pone.0318429.s005.docx]

**Reviewer #1:**

The manuscript investigated the correlation between chemotherapy treated elderly PC patients and HSM. A broad cohort has been studied with propensity matching strategy.

**Response:**

*Thank you for your detailed and insightful comments on our manuscript, "Chemotherapy and Heart-Specific Mortality in Elderly Men with Prostate Cancer: A Propensity Score Matching Analysis." We are truly grateful for the time and effort you have invested in reviewing our work and for the constructive feedback you have provided. Your suggestions are invaluable to us as we strive to refine and improve the quality of our research paper.*

*In response to your comments, we have carefully considered each point and have prepared a comprehensive, point-by-point reply. We have taken into account your recommendations for strengthening our methodology, clarifying our statistical analyses, and enhancing the clarity of our discussion. Our aim is to address all your concerns and to ensure that our manuscript meets the high standards expected in scientific publishing.*

*Please find our detailed response attached, and do not hesitate to contact us should you require any further clarification or additional information. We look forward to the opportunity to revise our manuscript with your guidance and to submit an improved version for consideration.*

*Once again, thank you for your support and expertise. We are hopeful that with your assistance, our paper will make a significant contribution to the field of oncology and cardiology research.*

Additional comments:

1. In the background section, the author did not include the most up-to-date data. Instead, the author wrote: “It is estimated that there will be 19.3 million new cancer cases in the world in 2020, with prostate cancer accounting for 7.4% of the total incidence ranking fourth, but only second to lung cancer in men [1]. In 2022, 268,490 new PC patients will be diagnosed in the United States, and 34,500 deaths from PC are expected, accounting for about 11% of male cancer deaths [2]. “ The prediction of the past years in 2020 and 2022 does not make sense in a manuscript submitted in 2024.

**Response:**

*Dear reviewer, thank you for your comments. Since this manuscript was written in 2021, there are some temporal biases. I'm sorry for our mistake. In the background section, we have updated the authoritative data for 2023 and 2024, and updated the references. The continued growth trend of prostate cancer still exists [1-2], so the views of our manuscript are not affected. We marked the modification in red font, hoping to meet your requirements, thank you!*

**Updated references:**

[1] Siegel RL, Miller KD, Wagle NS, Jemal A. Cancer statistics, 2023. CA Cancer J Clin. 2023 Jan; 73(1):17-48. doi: 10.3322/ caac.2173.pmid: 36633525.

[2] Siegel RL, Giaquinto AN, Jemal A. Cancer statistics, 2024. CA Cancer J Clin. 2024 Jan-Feb; 74(1):12-49. doi: 10.3322/ caac.21820.Epub 2024 Jan 17. Erratum in: CA Cancer J Clin.2024 Mar-Apr; 74(2):203. doi: 10.3322/ caac.21830.PMID: 38230766.

2. Some sentences are very confusing. I suggest the authors enhance the editing of the manuscript and ensure academic writing style is applied. For example in line 79-81: “Excluding breast and ovarian cancers, germline or somatic aberrations in DNA damage-repair genes that compromise genomic integrity are found in 19% of primary prostate cancers and nearly 23% of metastatic castration-resistant prostate cancer (mCRPC)”. Also in line 173-174: “It can be found that the higher the TNM stage and tumor histological grade, the more the number of chemotherapy." In addition, the “C” in section “conclusion” should be capitalized.

**Response:**

*Dear Reviewer, we sincerely apologize for the oversight in our manuscript and appreciate your valuable suggestions. We have revised the content from lines 79 to 81, where we intended to highlight the distinct treatment approaches for prostate cancer compared to solid tumors such as breast and ovarian cancer.*

*In the results section, specifically in the first paragraph discussing Table I, we have made comprehensive updates as follows: "It can be found that the higher the TNM stage and the tumor histological grade, the greater the number of patients receiving chemotherapy. PSA levels were higher in patients receiving chemotherapy. In addition, the proportion of patients receiving chemotherapy, who did not receive radiotherapy and who did not undergo surgery was higher."*

*In summary, we have updated our content to ensure clarity and have used red font to indicate the revised portions. We hope these changes meet your expectations. Thank you once again for your feedback.*

3. In the result section, the authors mentioned: “We used the inverse probability propensity weighted matching method to balance the influence of confounding factors, and the results showed that there was no significant difference in HSM between patients who received chemotherapy and those who did not (P=0.95) (Figure 5). However, what I see in Figure 5(B) is Kaplan-meier curve of PC patients with or without chemotherapy. The statement and the figure seem to be irrelavent.

**Response:**

*Dear Reviewer. Thank you for your comments. We apologize for the oversight and have recalculated the data based on the original sources. We have found that the results presented in our figures are indeed correct. However, we acknowledge that the conclusion may have been misinterpreted due to translation errors, leading to ambiguity in the text.*

*We have revised the description in the results section to align accurately with the data, and the updated conclusion is fully supported by our manuscript without any adverse impact on its content. We have highlighted the revised portions in red font to ensure clarity and to meet your expectations.*

*Thank you for your understanding and for the opportunity to address your concerns.*

4.The Figure 5B annotation is wrong. In the Plot, the chemotherapy yes (blue) is with lower risk but in the risk table the blue group is dropping out faster (higher risk)

**Response:**

*Dear Reviewer,*

*Thank you for your insightful comments. Upon your suggestion, we have conducted a thorough re-examination of Figure 5B. We have identified the issue you highlighted and have re-plotted the graph using R software. We discovered an erroneous definition within the R package that incorrectly assigned the colors below the labels to the opposite groups.*

*We have corrected this mistake, and the revised figure accurately reflects the data. We believe that our amendments address your concerns and meet the requirements.*

*We appreciate your guidance and are grateful for the opportunity to improve the quality of our manuscript.*

5. The patient stratification on chemotherapy seems too rough. For example, if the patient received a 1L chemo and then received other treatments like PARPi or ADT, and finally developed HSM. In this case, it is very hard to attribute HSM to chemotherapy. However, according to the method section, the authors would simply characterize patients with any LOT of chemotherapy as the chemotherapy group.

**Response:**

*Dear Reviewer,*

*Thank you for your comments. We apologize for the oversight and appreciate your feedback. As you rightly pointed out, the Surveillance, Epidemiology, and End Results (SEER) database only provides outcome data, and our analysis was retrospective in nature. Therefore, our statistical data only reveal whether patients received chemotherapy and their corresponding outcomes—survival or death. Consequently, it is indeed necessary to conduct further multicenter prospective clinical randomized controlled trials to validate our results. We have thoroughly described the limitations of our manuscript in the last paragraph of the discussion section.To facilitate your review, we marked the beginning of the paragraph in red font. I hope our explanation can meet your requirements.*

*We are actively conducting prospective multicenter studies and establishing relevant disease-specific databases. In the near future, we will provide further explanation and validation in new publications. We hope our explanation meets your requirements. Thank you!*

**Reviewer #2:**

1. Technical Soundness and Data Support: The manuscript is technically sound, and the data presented effectively support the conclusions. The use of the SEER database to extract patient information is a strong methodological choice, and the study appropriately captures the relevant patient population. The multivariate logistic regression analysis and competing risk model are well-utilized to demonstrate the relationships between chemotherapy and heart-specific mortality (HSM) among elderly prostate cancer patients. Your findings that chemotherapy does not increase HSM and that it can have beneficial long-term outcomes are both important and relevant for clinical decision-making.

2. Statistical Analysis: The statistical analysis has been performed rigorously and appropriately. The use of multivariate logistic regression, propensity score matching, and the competing risk model is highly commendable and well-suited for controlling potential confounders and exploring the treatment effects. The matching process ensures comparability between the chemotherapy and non-chemotherapy groups, which adds robustness to your conclusions. The results are presented clearly, with appropriate statistical measures, such as hazard ratios (HR) and p-values, to substantiate your findings.

3. Data Availability: The manuscript makes use of the SEER database, which is publicly available, ensuring transparency and reproducibility of the findings. You have appropriately referenced the SEER database, and it appears that all data necessary to replicate the study are accessible. This is in line with the journal's requirements for making underlying data fully available, and it greatly enhances the credibility of your study.

4. Presentation and Language: The manuscript is presented in an intelligible fashion, and the standard of English is appropriate for a scientific audience. The abstract and main text are clear, and the methods, results, and conclusions are logically structured and easy to follow. There are no noticeable grammatical issues, and the writing effectively communicates the key findings and implications of the research.

**Response:**

*Dear Reviewer,*

*We are deeply honored that you have taken the time out of your busy schedule to review our manuscript and provide such a positive evaluation. Your recognition and encouragement are not only an affirmation of our past work but also a wellspring of motivation for us to continue exploring the unknown and striving for excellence. We are well aware that the path of scientific research is full of challenges and hardships, but every word of encouragement from you is like the warm sun in spring, warming our hearts and filling us with confidence and anticipation for future research.*

*We are particularly grateful for points 1 to 4 that you mentioned in your comments. These specific and constructive suggestions not only show us our shortcomings but also point the way forward. The passion for science and attention to detail revealed in your words have greatly inspired us. We believe that these valuable pieces of feedback will become an indispensable guide in our subsequent research, helping us to more accurately identify problems and more efficiently advance our research process.*

*Once again, we express our sincere gratitude to you. Thank you for your attention to our work and for your valuable opinions. We look forward to incorporating these suggestions into our research to achieve higher academic accomplishments. At the same time, we also look forward to continuing to receive your guidance and assistance in the future.*

5. General Comments:

The abstract clearly outlines the objective, methods, results, and conclusions of the study, which provides a concise and effective summary for readers.

The use of a competing risk model to differentiate between HSM and other cause mortality (OCM) is particularly valuable and demonstrates a nuanced understanding of the potential confounders associated with elderly patients.

The discussion appropriately addresses the clinical implications of the findings, especially in regard to the benefits of chemotherapy in elderly prostate cancer patients despite concerns about cardiotoxicity.

It may be helpful to include a brief discussion on the potential mechanisms through which chemotherapy could provide long-term survival benefits in this patient population, even in the presence of cardiotoxicity risks. Adding a few references that explore these mechanisms might provide additional depth to your discussion.

**Response:**

*Dear Reviewer,*

*We would like to express our gratitude for your insightful suggestion in point 5 of your review. Heeding your advice to "add references that explore these mechanisms to provide additional depth to your discussion," we have conducted a thorough literature review of advancements in prostate cancer and chemotherapy research over the past decade. Consequently, we have added a new paragraph in the discussion section to address the content you recommended:*

*"Prostate cancer is a prevalent malignant tumor in men, and chemotherapy plays a significant role in its treatment, particularly in the management of castration-resistant prostate cancer (CRPC). Currently, the potential mechanisms by which chemotherapy contributes to long-term survival benefits for patients with prostate cancer (PC) are as follows: 1) Anti-mitotic effects: Chemotherapeutic agents such as docetaxel and cabazitaxel inhibit microtubule depolymerization, preventing cancer cell mitosis and thereby suppressing tumor cell proliferation. This anti-mitotic action is a direct mechanism by which chemotherapeutic drugs extend survival [1]. 2) Downregulation of androgen receptor (AR) activity: Docetaxel not only inhibits microtubule depolymerization but also downregulates the transcriptional activity of AR, suppresses AR gene expression, and inhibits AR activity by increasing FOXO1 levels [2]. This mechanism helps maintain therapeutic effects in castration-resistant prostate cancer [3]. 3) Promotion of apoptosis: Chemotherapeutic agents like docetaxel have anti-Bcl-2 and anti-Bcl-XL properties, exerting anti-tumor effects by promoting apoptosis [4]. The increase in apoptosis helps reduce tumor burden, potentially extending patient survival [5]. In addition to the three main mechanisms mentioned above, the anti-angiogenic and immunomodulatory effects of chemotherapeutic drugs should not be overlooked [6-7]. These effects make chemotherapy play a crucial role in PC, especially in CRPC, effectively prolonging patient survival."*

*For your convenience in reviewing, we have highlighted the addition in blue font within the text. We hope that our supplementation meets your expectations. Thank you!*

**References**

[1]Di Lorenzo G, DʼAniello C, Buonerba C, Federico P, Rescigno P, Puglia L, Ferro M, Bosso D, Cavaliere C, Palmieri G, Sonpavde G, De Placido S. Peg-filgrastim and cabazitaxel in prostate cancer patients. Anticancer Drugs. 2013 Jan; 24 (1) : 84-9. Doi: 10.1097 / CAD. 0 b013e32835a56bc. PMID: 23044721.

[2]Fitzpatrick JM, de Wit R. Taxane mechanisms of action: potential implications for treatment sequencing in metastatic castration-resistant prostate cancer. Eur Urol. 2014 Jun; 65 (6) : 1198-204. The doi: 10.1016 / j. ururo. 2013.07.022. Epub 2013 Jul 25. PMID: 23910941.

[3]Darshan MS, Loftus MS, Thadani-Mulero M, Levy BP, Escuin D, Zhou XK, Gjyrezi A, Chanel-Vos C, Shen R, Tagawa ST, Bander NH, Nanus DM, Giannakakou P. Taxane-induced blockade to nuclear accumulation of the androgen receptor predicts clinical responses in metastatic prostate cancer. Cancer Res. 2011 Sep 15; 71(18):6019-29. doi: 10.1158/0008-5472.CAN-11-1417.Epub 2011 Jul 28.PMID: 21799031; PMCID: PMC3354631.

[4]Beltran H, Beer TM, Carducci MA, de Bono J, Gleave M, Hussain M, Kelly WK, Saad F, Sternberg C, Tagawa ST, Tannock IF. New therapies for castration-resistant prostate cancer: efficacy and safety. Eur Urol. 2011 Aug; 60 (2) : 279-90. The doi: 10.1016 / j. ururo. 2011.04.038. Epub 2011 May 4. PMID: 21592649.

[5]Rahman R, Rahaman MH, Hanson AR, Choo N, Xie J, Townley SL, Shrestha R, Hassankhani R, Islam S, Ramm S, Simpson KJ, Risbridger GP, Best G, Centenera MM, Balk SP, Kichenadasse G, Taylor RA, Butler LM, Tilley WD, Conn SJ, Lawrence MG, Wang S, Selth LA. CDK9 inhibition inhibits multiple oncogenic transcriptional and epigenetic pathways in prostate cancer. Br J Cancer. 2024 Oct; 131(6):1092-1105. doi: 10.1038/ s41416-024-02810-8.Epub 2024 Aug 8.PMID: 39117800; PMCID: PMC11405875.

[6]Lohiya V, Aragon-Ching JB, Sonpavde G. Role of Chemotherapy and Mechanisms of Resistance to Chemotherapy in Metastatic Castration-Resistant Prostate Cancer. Clin Med Insights Oncol. 2016 Oct 9; 10(Suppl 1):57-66. doi: 10.4137/ CMO.S34535.PMID: 27773999; PMCID: PMC5065075.

[7]Guo C, Crespo M, Gurel B, Dolling D, Rekowski J, Sharp A, Petremolo A, Sumanasuriya S, Rodrigues DN, Ferreira A, Pereira R, Figueiredo I, Mehra N, Lambros MBK, Neeb A, Gil V, Seed G, Terstappen L, Alimonti A, Drake CG, Yuan W, de Bono JS; International SU2C PCF Prostate Cancer Dream Team. CD38 in Advanced Prostate Cancers. Eur Urol. 2021 Jun; 79 (6) : 736-746. The doi: 10.1016 / j. ururo. 2021.01.017. Epub 2021 Mar 5. PMID: 33678520; PMCID: PMC8175332.

This manuscript makes a significant contribution to the literature on the management of prostate cancer in elderly patients. It provides evidence that challenges the notion that chemotherapy necessarily increases heart-specific mortality, suggesting that chemotherapy can offer survival benefits. The study is methodologically sound, the statistical analyses are well-executed, and the findings are presented in a clear and coherent manner. I accept the publication of this work in PLOS ONE.

**Reviewer #3:**

The authors used the SEER database to compare the mortality rate from cardiovascular events in prostate cancer patients who received chemotherapy or not.

I understand that propensity score matching is a useful statistical method. I think that patients who received chemotherapy may have had confounding factors related to the occurrence of cardiovascular events, such as diabetes, past cardiovascular events, or a history of hypertension.

I think it would be a useful report if the database could be used to investigate such factors, including past medical conditions.

**Response:**

*Dear Reviewer,*

*We extend our sincere gratitude for your invaluable comments. As you rightly pointed out, the concerns you've raised are indeed at the forefront of our team's attention. However, given that our study relied on the SEER database, which only provides outcome data, and considering the retrospective nature of our analysis, these factors have indeed limited the scope of information we could obtain. Specifically, we were only able to observe, based on existing statistical data, whether patients had received chemotherapy and analyze its potential impact on patient survival or death outcomes.*

*In this context, we are keenly aware of the necessity to further validate our research findings. Consequently, we strongly recommend and look forward to conducting multicenter, prospective clinical randomized controlled trials to more comprehensively and deeply explore the impact of chemotherapy on patient outcomes, thereby optimizing our research conclusions.*

*Additionally, we have detailed the limitations of our current study and potential directions for future research in the last paragraph of the discussion section. We firmly believe that through continued research and exploration, we can get closer to the truth and provide patients with more effective treatment plans.*

*For your convenience in reviewing, we have highlighted the corresponding paragraphs with a yellow background, hoping for your understanding. Furthermore, we have actively engaged in prospective multicenter research and successfully built a specialized disease database. In the near future, we plan to provide a more detailed explanation and validation in an upcoming publication. We look forward to meeting your expectations and requirements through our exposition and thank you again for your comments!*

**Reviewer #4:**

The study presents a significant investigation into the relationship between chemotherapy and heart-specific mortality (HSM) in elderly prostate cancer (PC) patients. The use of a large dataset from the SEER enhances the reliability of the findings. The conclusions drawn from the analysis are both and timely, given the increasing incidence of prostate cancer and the importance of understanding treatment implications on overall health outcomes.

Strengths

1. Large Sample Size: The inclusion of 135,183 elderly prostate cancer patients strengthens the statistical power of the study, allowing for more robust conclusions.

2. Methodological Rigor: The use of multivariate logistic regression and propensity score matching to control for confounding variables is commendable. This approach enhances the validity of the results by minimizing biases.

3. Clear Objectives: The study's aim to assess the impact of chemotherapy on HSM is clearly stated, making it easy for readers to understand the focus of the research.

4. Significant Findings: The finding that HSM is higher in non-chemotherapy patients has important clinical implications and suggests that chemotherapy may provide a protective effect in terms of heart-specific mortality.

**Response:**

*Dear Reviewer,*

*Thank you immensely for your insightful comments, valuable suggestions, and the opportunity to refine our manuscript. Your time in reviewing our responses and paper is greatly appreciated. We hold you in high esteem as a kind-hearted and renowned academic reviewer who has generously offered numerous excellent ideas. Consequently, upon receiving your email feedback, we promptly embarked on revising the manuscript. We have diligently addressed your suggestions, incorporating the comments below and providing detailed, point-by-point responses.*

Areas for Improvement

1. Clarification of Chemotherapy Types: The abstract does not specify the types of chemotherapy administered. Providing details on the regimens used could help contextualize the findings and their applicability to different treatment protocols.

**Response:**

*Dear Reviewer,*

*Thank you for your insightful comments. We appreciate your recognition of the strengths of the SEER database used in our study, such as its objectivity and large sample size. You are correct in pointing out that one of the limitations of the SEER database is the lack of detailed information on specific chemotherapy regimens. The database only provides two indicators for chemotherapy classification, as shown in the figure below, and we have included the relevant annotation files in the supplementary materials as attachments.*

*To address this limitation and to further substantiate our findings, we have initiated prospective multicenter studies and have established a specialized disease database. Our database contains comprehensive data on the chemotherapy cycles and regimens for all patients, which we believe will allow us to provide a more detailed explanation and validation in future publications.*

*We have highlighted the sections in our response that correspond to the areas of concern you raised, and we hope that our explanation and the steps we are taking to address this issue meet your expectations.*

2. Discussion of Limitations: While the study mentions confounders, a more detailed discussion of potential limitations, such as selection bias or the influence of unmeasured confounders, would strengthen the overall rigor of the study.

**Response:**

*Dear Reviewer,*

*Thank you for your valuable feedback. As suggested, we have significantly expanded the discussion of the potential limitations of our study in the final paragraph of the discussion section to enhance the overall rigor of the research. To facilitate your review, we have highlighted the newly added discussion in green font. We hope that this additional information meets your expectations.*

*Thank you again for your guidance and for providing us with the opportunity to improve our manuscript.*

3. Long-term Follow-up: It would be beneficial to discuss the follow-up duration for assessing HSM and other causes of mortality. Longer follow-up periods may yield different insights into the long-term effects of chemotherapy.

**Response:**

*Dear Reviewer,*

*Thank you very much for your insightful comments. Your suggestions are indeed correct and can effectively help us further enhance the quality of our manuscript. However, for the following reasons, we have only collected PC data from after 2010:*

*a. In 2010, the International Union for Cancer Control (UICC) and the American Joint Committee on Cancer (AJCC) jointly published the seventh edition of the TNM staging system for prostate cancer, which underwent significant changes compared to the sixth edition. To ensure the consistency of the data collected, our study began to include cases from 2010 onwards.*

*b. In 2010, the guidelines for the diagnosis and treatment of prostate cancer in several countries no longer recommended the use of prostate-specific antigen (PSA) testing for routine prostate cancer screening. This is because, although PSA screening has increased the detection rate of prostate cancer, it may also lead to the overdiagnosis and overtreatment of indolent prostate cancer, thereby affecting the quality of life of patients and causing a waste of medical resources. The results of the Prostate, Lung, Colorectal, and Ovarian Cancer Screening Trial (PLCO) conducted by the National Cancer Institute in the United States and the European Randomized Study of Prostate Cancer Screening (ERSPC) have influenced this decision.*

*c. Significant advancements in treatment: In 2010, the U.S. FDA approved Sipuleucel-T (Provenge) for the treatment of metastatic prostate cancer patients who have failed castration therapy. Sipuleucel-T is a dendritic cell (DC) vaccine created by fusing tumor-specific antigens with GM-CSF to sensitize DCs. It is the first therapeutic vaccine approved by the U.S. FDA and holds significant clinical importance.*

*d. Approval of new endocrine therapies and chemotherapy drugs: In 2010, the U.S. FDA also approved Cabazitaxel (Jevtana) in combination with prednisone for the treatment of hormone-resistant prostate cancer after docetaxel, a first-line chemotherapy, has failed. Additionally, Abiraterone, an oral inhibitor of the cytochrome P450 enzyme CYP17, was reported at the 2010 ESMO Congress to have significant effects in treating advanced prostate cancer.*

*These developments and changes signify that the diagnosis and treatment of prostate cancer entered a new era in 2010, with new understandings in screening and diagnosis, as well as significant progress in treatment, providing patients with more treatment options and better therapeutic outcomes. Therefore, our study began collecting data from 2010. We will continue to follow up with these patients at a later date to achieve long-term follow-up. We hope our explanation meets your requirements. Thank you!*

4. Patient Characteristics: Additional information on the baseline characteristics of patients who received versus did not receive chemotherapy (e.g., comorbidities, performance status) would provide a clearer picture of the study population.

**Response:**

*Dear Reviewer,*

*Thank you for your valuable feedback. We have thoroughly presented the baseline characteristics of patients who did and did not receive chemotherapy before and after propensity matching in Table 1. However, as previously mentioned, the SEER database does not include variables such as comorbidities and performance status. Therefore, we have not been able to describe these two characteristics in Table 1 as you suggested.*

*In the discussion of the limitations of our study, we have also delved into this issue, stating, "The SEER database lacks patient comorbidity data. Although propensity score matching was used to exclude the interference of confounding factors, it is still not possible to rule out the inherent impact of comorbid factors on outcomes."*

*We hope that our explanation meets your requirements. Thank you once again for your understanding and guidance.*

Conclusion

Overall, this study contributes valuable insights into the relationship between chemotherapy and heart-specific mortality in elderly prostate cancer patients. The findings suggest that chemotherapy may not only be safe but potentially beneficial for long-term survival in this population. Addressing the suggested improvements could enhance the clarity and impact of the research.

**Response:**

*Dear Reviewer,*

*We would like to express our heartfelt gratitude once again for the valuable suggestions you have provided regarding our study. Your input has been instrumental in enhancing the quality of our manuscript.*

*Should you have any further questions or require additional clarification regarding our paper, please do not hesitate to let us know. We are confident that our responses and revisions will meet with your satisfaction.*

*Thank you again for your support and assistance.*
