# Supplementary material for: Chemotherapy and Heart-Specific Mortality in Elderly Men with Prostate Cancer: A Propensity Score Matching Analysis
Source: PLoS One. 2025 Apr 11;20(4):e0318429. doi: 10.1371/journal.pone.0318429 (PMC11990641; doi:10.1371/journal.pone.0318429)
Supplement: Attachment — Submitted filename: Response_to_Reviewers_auresp_2.docx [file pone.0318429.s006.docx]

**Reviewer #1 and Editor:**

Reviewer #1: The authors have addressed my concerns. Some of my concerns were related to the cohort selection and categorization, and the authors acknowledged the limits in the discussion session.

Editor notes: Some of the concerns proposed by reviewer #1 were about the cohort selection and categorization, but you just discussed these limitations instead of performing further analysis and changes. Thus, actually, I think the statistical part can be considered to be further clarified and improved to avoid potential flaw.

**Response:** Thank you for your comments. At present, based on the limitations of data collection in the SEER database, we do not have a better way to further modify and optimize for question 5. Because this is an inherent defect of the database, all published papers related to the SEER database have the same limitation. Therefore, we fully discuss it in the limitations section of the discussion.

As in Reviewer 1's response, our interpretation has addressed reviewer 1's concerns. However, as the editor noted (underlined), there was no way to further optimize cohort classification on the basis of the SEER database. However, we fully explain in the limitations section why there is no way to further optimize. In addition, we have been collecting multi-center data in combination with a number of institutions. In the future, we will further analyze and verify the data of accurate chemotherapy regimens and specific courses, as well as comorbidities collected by ourselves. We hope our answers can meet your requirements. Thanks again for your advice!

**Reviewer #3:**

I feel that the authors' corrections are insufficient.

I hope that they will add further consideration.

Editor notes: Please consider to further address the previous feedbacks of this expert.

**Response:**

Dear reviewer,

We greatly appreciate your review of this study and your valuable comments. You correctly pointed out the importance of considering potential confounding factors when assessing cardiovascular event mortality in prostate cancer patients who received chemotherapy versus those who did not. We agree that these factors may have influenced the results and controlled for these potentially confounding variables in the analyses.

In this study, we used the SEER database for propensity-score matching (PSM), a statistical method designed to reduce the effects of selection bias in observational studies. Our goal was to create two patient populations that were similar in baseline characteristics so that we could more accurately compare mortality from cardiovascular events between patients who received chemotherapy and those who did not.

In response to your concerns, we did account for multiple confounders including diabetes, previous cardiovascular events, and history of hypertension in our analyses. The SEER database provided this key demographic and clinical information, allowing us to include these variables in the propensity score model. Specifically, we adjusted for the following variables:

**Age:** Consider that age is a major risk factor for prostate cancer and cardiovascular disease.

**Ethnicity:** Different ethnic groups may have different disease rates and treatment patterns.

**Gender**: Although this study was limited to men, gender may be a relevant factor in other types of cancer.

Cardiovascular events (" **Diseases of Heart** ") : our propensity matching used this term combined with "Dead (attributable to causes other dx)" in the outcome factor to confirm the number of deaths due to cardiovascular events. Therefore, this term is very important and is also highlighted in our study.

"**Diabetes Mellitus**" : Diabetes mellitus is an important risk factor for cardiovascular disease, however, unfortunately, data on diabetes comorbidity were only available for 13 individuals after propensity matching. We believe that such an analysis may cause serious bias; therefore, we do not present the results of this part.

"**Hypertension without Heart Disease**" : Hypertension is another known risk factor for cardiovascular disease. Unfortunately, there are a large number of patients with comorbidity of Hypertension and Heart Disease. Therefore, after further mining of the database, we found an entry "Hypertension without Heart Disease". Unfortunately, we found this number to be only eight and therefore did not conduct further analysis. See the following picture for specific terms:

By including these variables in the propensity score model, we were able to match patients who were similar in these key factors, thereby reducing the effect of confounding factors on the results. More important, however, the SEER database does not know the sequence of these events in terms of illness or chemotherapy receipt, and some important programs may have had insufficient numbers for the analysis because of data collection anomalies. Therefore, we did not analyze or present other important comorbidity data.

However, with a large sample size and with minimal bias avoidance, our results show that there is no significant difference in cardiovascular mortality between patients who received chemotherapy and those who did not, after adjustment for these potential confounders.

We understand that despite our measures, there are certain limitations associated with any observational study. We agree that further studies, particularly prospective, well-designed studies, will help validate our findings and may provide more insight into the effects of chemotherapy on cardiovascular health in prostate cancer patients.

Thank you again for your valuable comments. We believe that by considering these confounding factors, we will have a more accurate understanding. We have been collecting multi-center data in combination with several institutions, and we will further analyze and verify it by collecting accurate comorbidity data ourselves in the future. We hope our answers can meet your requirements. Thanks again for your advice!
